# Comparative Study of Aqueous Acid–Base Properties of Tungstocene and Molybdocene Complexes

**DOI:** 10.1021/acs.organomet.5c00345

**Published:** 2025-12-12

**Authors:** Niklas Stix, Miljan Z. Ćorović, Sophia S. Schiller, Antoine Dupé, Nadia C. Mösch-Zanetti

**Affiliations:** Institute of Chemistry, Inorganic Chemistry, University of Graz, 8010 Graz, Austria

## Abstract

The aqueous chemistry of molybdenum and tungsten complexes is relevant due to their occurrence in metalloenzymes. However, water-stable and -soluble model complexes in biologically relevant higher oxidation states are rare. Metallocenes of the type [Cp_2_M­(OH_2_)]^2+^ (M = Mo, W) exhibit such properties despite the nonbiomimetic cyclopentadienyl (Cp) ligand. Therefore, the aqueous acid–base properties of these bis-aqua tungstocenes and molybdocenes were investigated, as their +IV oxidation states and coordinated H_2_O render them ideal candidates. The precursors [Cp_2_MoCl_2_] (**1a**), [Cp_2_Mo­(μ-OH)_2_MoCp_2_]­(*p*TsO)_2_ (**1b**), [Cp_2_Mo­(*p*TsO)_2_] (**3**) and their tungsten analogues [Cp_2_WCl_2_] (**2a**), [Cp_2_W­(μ-OH)_2_WCp_2_]­(*p*TsO)_2_ (**2b**), and [Cp_2_W­(*p*TsO)_2_] (**4**) were studied via aqueous potentiometric titrations. Molybdocene acted as a triprotic acid, with deprotonation occurring from both mono- and dimeric species, while tungstocene reacted as a diprotic acid exclusively in its monomeric form. Tungsten complexes exhibit higher acidity with 1–1.5 lower p*K*_a_ values than molybdenum, which is of general interest for the understanding of tungstoenzymes. The substitution of chlorides by tosylates allowed the isolation of **3** and **4**, which proved to be less suitable precursors for aqueous chemistry, as they were more difficult to hydrolyze, leading to partial degradation upon hydrolysis. This indicates that simply the presence of a Cp_2_M motif is not sufficient for the formation of the aqueous species.

## Graphical abstract

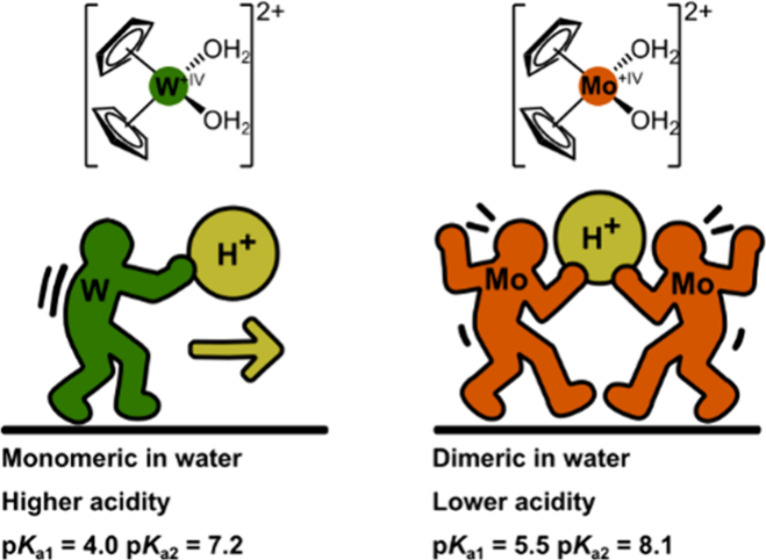

## Introduction

Water is the solvent of life and plays a crucial role in biological systems, serving not only as a solvent but also as a substrate in various enzymatic reactions. In organometallic chemistry, water is rarely used due to the usually high sensitivity of the M–C bond toward hydrolysis. However, it has unique properties, such as a high boiling point, polarity, and ability to form hydrogen bonds, which make it an attractive medium for both ecological and economic reasons.^1^ In metalloenzymes, the presence of coordinated water is often pivotal, as it can participate in catalytic cycles and frequently undergo deprotonation to facilitate reaction mechanisms. This is also the case in molybdo- and tungstoenzymes, a class of redox enzymes that catalyze the transfer of an oxygen atom to or from a substrate and where water is often the source of oxygen.^2,3^ For example, in the xanthine oxidase family of molybdoenzymes, a water molecule is coordinated and deprotonated alongside the oxidation of the metal center.^4^ Also, sulfite oxidase requires a water molecule,^5^ and for DMSO reductases, water is a product.^2^ Similar involvement of water is also found in related tungstoenzymes.^6^ Despite their chemical similarity, molybdenum and tungsten cannot be directly exchanged in the active centers of enzymes without significantly affecting their activity.^7^ Any differences between the reactivity of tungstoenzymes compared to the respective molybdoenzymes are generally attributed to the more negative redox potential of W^VI^/W^IV^ compared to that of the Mo^VI^/Mo^IV^ redox couple.^2,8,9^ This could be explained by relativistic effects, which are stronger for the higher homologue W compared to Mo.^10^ Therefore, exchanging Mo with W in the redox molybdo- and tungstoenzymes is expected to exhibit a significant influence. Among this group of enzymes with common structural features, there is a single example, the *acetylene hydratase* (AH), which does not catalyze a redox reaction but rather the hydration of acetylene.^11^ It is therefore noteworthy that the exchange of W by Mo in AH leads to reduced activity.^12^ While its mechanism is as yet unclear, the molecular structure was determined by X-ray crystallography, which pointed toward a water molecule coordinated to the W­(IV) active site.^6,13^ Therefore, understanding the aqueous-phase speciation of Mo and W in biologically relevant oxidation states is essential for interpreting their behavior in biological environments. Since such metal centers are also known to form polyoxometalates, their speciation study is of high interest for a wide range of researchers due to the diverse applications of the latter in, e.g., biological and medical fields or in homogeneous catalysis.^14^ Furthermore, speciation studies deliver p*K*_a_ values of metal aqua compounds, which may be used as a measure for their Lewis acidity even in solvents other than water.^15^

Model complexes for AH allowing speciation require a metal in the oxidation state + IV and should be soluble and stable in water. This excludes many known Mo and W complexes that were developed for studies to model biological pyranopterin active sites. However, metallocenes of the type [Cp_2_M­(OH_2_)]^2+^ (M = Mo, W) are known to exhibit such properties despite the nonbiomimetic, organometallic cyclopentadienyl (Cp) ligand. The two aqua ligands allow for water solubility, while also enabling diverse reactivity. Therefore, their speciation is also of interest for many research studies in organometallic chemistry due to the widespread use of the Cp platform.

Molybdocenes of the type [Cp_2_MoCl_2_] (**1a**) and [Cp_2_Mo­(μ-OH)_2_MoCp_2_]^2+^ (**1b**) have been extensively studied for their speciation properties and reactivities in water.^16–31^ They demonstrate catalytic activity in various reactions, including the hydrolysis of phosphoesters, carboxylic acid esters, and vinyl ethers, catalytic nitrile hydration, transfer hydrogenation, and H/D exchange.^21,22,25^ In contrast, analogous tungsten complexes remain widely unexplored. While the tungstocene motif [Cp_2_W]^2+^ is generally well-known to bind diverse ligands, such as in [Cp_2_WCl_2_] (**2a**), [Cp_2_WO], and [Cp_2_WH_2_],^32–36^ their speciation properties in water are far less explored. The only aqueous tungstocene that has been described is the μ-hydroxy bridged complex [Cp_2_W­(μ-OH)_2_WCp_2_]^2+^ (**2b**).^37,38^ However, water was utilized only for its synthesis and never as a solvent for further reaction.

Aqueous speciation properties of molybdocenes were investigated by the group of Tyler using extensive titration and ^1^H NMR studies, which supports acid–base equilibria occurring in water, as shown in Scheme 1.^24^ Thereby, the p*K*_a_ values were determined, which is of high importance, since it was found that hydrolysis reactions catalyzed by molybdocenes show an activity optimum at pH = 7.^21,25^ Thus, such equilibria and p*K*_a_ data allow for statements on the nature of the catalytically active species.

**Scheme 1:**
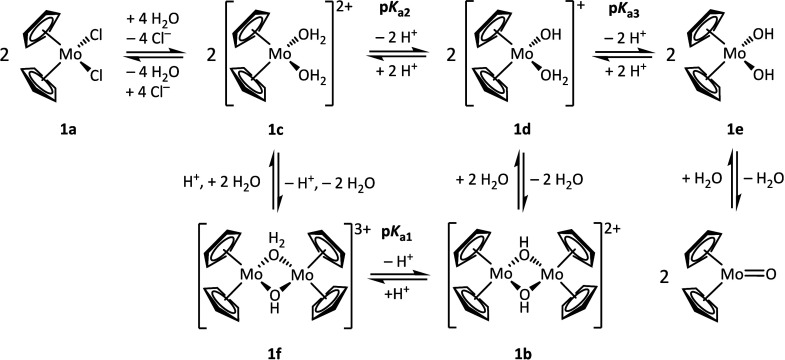
Acid–Base Equilibria Suggested by Tyler and Co-Workers for the [Cp_2_Mo]^2+^ Moiety Dissolved in Water^24^

Here, we present our titration studies of the analogous tungstocene complexes [Cp_2_WCl_2_] (**2a**) and [Cp_2_W­(μ-OH)_2_WCp_2_]­(*p*TsO)_2_ (**2b**), revealing different acid–base equilibria and more acidic p*K*_a_ values compared to the molybdocenes.

## Results and Discussion

### Synthesis of Metallocenes

The preparations of the here investigated compounds [Cp_2_MCl_2_] (M = Mo **1a**, W **2a**) and [Cp_2_M­(μ-OH)_2_MCp_2_]­(*p*TsO)_2_ (M = Mo **1b**, W **2b**) were previously described.^16,32,33,38^ We used slightly modified literature procedures, such as omitting the isolation of intermediates and modification of cleanup procedures. **1a** and **2a** were directly crystallized from the respective reaction solutions and washed with pentane before drying in vacuo. For **2a**, suitable single crystals for the X-ray diffraction analysis were obtained. A molecular view is displayed in Figure S22, and crystallographic data and selected bond lengths and angles are given in Tables S3–S5. The structure exhibits distorted tetrahedral geometry and is overall similar to the previously reported structure for **1a**.^39^ Compounds **1b** and **2b**, respectively, were isolated by extraction with MeOH and washing with Et_2_O.

### Titration of Aqueous Molybdocene

For comparative reasons and to establish identical conditions of investigation, we performed potentiometric studies not only with the tungstocene but also with the molybdocene complexes, the latter having been investigated previously by Tyler and co-workers.^24^ The dichlorido molybdocene (**1a**) or the dinuclear hydroxido molybdocene (**1b**), respectively, was dissolved in water, and the reaction mixtures were acidified with 1.2 M hydrochloric acid to pH = 2. These solutions were titrated with 0.01 M NaOH up to a pH of 11, as described in the Supporting Information. According to Scheme 1, after the acidification of **1a** or **1b**, a mixture of [Cp_2_Mo­(μ-OH)­(H_2_O)_2_MoCp_2_]^3+^ (**1f**) and [Cp_2_Mo­(H_2_O)_2_]^2+^ (**1c**) should be present. Our titration curves are shown in Figure 1. In accordance with the literature data, we observed three equivalence points (EPs). The EPs represent the values at which a species has been completely deprotonated by the titrant. In the obtained titration curves, the EPs are found at the same pH values for both starting complexes **1a** and **1b**, supporting the fact that the same species are present during the titration. However, we noticed a significant difference between the titrations of **1a** and **1b**, respectively. In an ideal titration curve of a triprotic acid, the amount of titrant needed to titrate from EP1 to EP2 would be one equivalent of base (Δ­(EP1,2) = 1). The same would then be expected when titrating further from EP2 to EP3 (Δ­(EP2,3) = 1) (see Figure S1 for visualization). This is observed for **1a** (Table 1, Δ­(EP1,2) 1.01; Δ­(EP2,3) 0.89), with both values being close to 1. In contrast, for compound **1b**, Δ­(EP1,2) is 0.56, which is significantly lower than the expected value of 1, while Δ­(EP2,3) with 0.83 is closer to the expectation. Note that **1b** is a dimer that produces two monomers according to Scheme 1. Therefore, the values of the ΔEPs are given per metal center to eliminate the difference between the monomeric and dimeric species. The different values of Δ­(EP1,2) obtained after basic titration of **1a** and **1b** suggest that the ratios of **1f** vs **1c** are different in the two starting materials. Thus, after acidifying to pH 2, compound **1a** is mainly converted into **1c** while little **1f** is formed. In contrast, at this low pH, **1b** is protonated into **1c** and **1f** in an approximate ratio of 1:1. This is presumably due to the higher acidic property of dichlorido complex **1a** vs dimer **1b** (aqueous solution before the titration of **1a**, pH 3–4, and **1b**, pH 5.5–6.5). Under these highly acidic conditions, **1a** forms predominantly **1c** because the equilibrium between **1d** and **1b** requires a higher pH. Finally, since dimerization does not play a role in the formation of **1e**, the titration curves at pH values >7 are identical for both starting materials (Figure 1). This suggests that the molybdocene does not act as a regular triprotic acid but rather initially as two different diprotic acids (the monomer **1c** and the dimer **1f**), which converge to **1e** at higher pH values.

**Figure 1:**
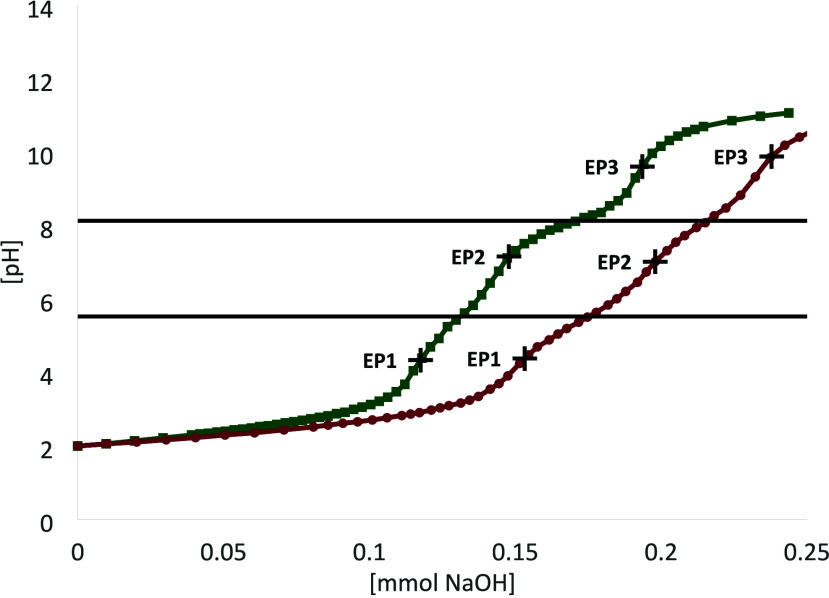
Titration curves of **1a** (red circles) and **1b** (green squares). The data points shown represent each measurement point. Solutions of **1a** and **1b** were acidified to a pH of 2 and then titrated with 0.01 M NaOH solution. The calculated p*K*_a_ values are given as black lines. EPs are given as black + symbols.

**Table 1:** Base Equivalents Required between EPs Relative to the Metal Centers^a^

precursor complex	**Δ(EP1,2)**	**calc.** value	**Δ(EP2,3)**	**calc.** value
**1a**	1.01 ± 0.05	1	0.89 ± 0.15	1
**1b**	0.56 ± 0.12	1	0.83 ± 0.05	1
**1a reverse**	0.57	1	0.96	1
**1b reverse**	0.50	1	1.0	1
**2a**	1.08 ± 0.10	1		
**2b**	1.06 ± 0.11	1		

a The base equivalents that were added during titrations to progress from one EP to the next. Values are the average of 4 measurements with the standard deviation, except for the reverse titrations. These were only measured once to confirm the findings from the basic titrations.

Our titrations allow the determination of the p*K*_a2_ and p*K*_a3_ values, being 5.51 and 8.12, respectively, which are very close to the literature data (5.6, 8.3). p*K*_a1_ could not be determined due to its low value and due to the above-described equilibrium shifts.

Again, since dimerization does not play a role at high pH, we wondered whether acidic titration of **1a** or **1b**, respectively, starting from a high pH, would lead to identical curves. Thus, aqueous solutions of **1a** and **1b** were adjusted to pH 11 by the addition of 1 M NaOH and titrated with 0.01 M HCl. The resulting titration curves are virtually identical, as shown in Figure 2. The numerical base equivalents needed between the EPs reveal that the protonation leads to **1d,** which is in equilibrium with **1b** (ratio approximately 1:1), and they are both protonated stepwise according to the p*K*_a_ values. The determined p*K*_a_ values are identical within experimental error to those obtained by basic titration (Tables 1 and 2). This demonstrates that the basic titration of **1a** (Figure 1) is different because the monomer–dimer equilibrium between **1c** and **1f** lies largely on the side of **1c**.

**Figure 2:**
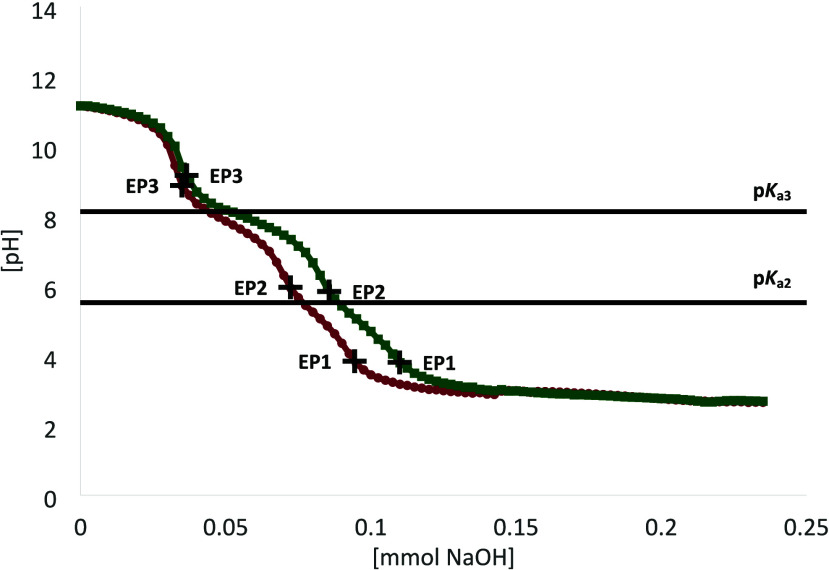
Titration curves of **1a** (red circles) and **1b** (green squares). The shown data points represent each measurement point. Solutions of **1a** and **1b** were set to a pH of 11 and then titrated with 0.01 M HCl solution. The calculated p*K*_a_ values are given as black lines. EPs are given as black + symbols.

**Table 2:** p*K*_a_ Values for the Different Precursor Complexes^a^

precursor complex	**p** *K*_**a2**_	**p** *K*_**a3**_
**1b**	5.78 ± 0.16	8.20 ± 0.07
**1a**	5.55 ± 0.07	8.23 ± 0.02
**1b reverse**	4.84	7.81
**1a reverse**	4.99	7.67
**1a + 1b**	5.51 ± 0.33	8.12 ± 0.19
**2b**	3.77 ± 0.16	7.06 ± 0.08
**2a**	4.46 ± 0.11	7.28 ± 0.05
**2a + 2b**	3.99 ± 0.35	7.15 ± 0.13

a Values are given as the average of 4 measurements with the standard deviation, except for the reverse titrations. These were only measured once to confirm the findings from the basic titrations.

Our results are highly relevant for catalytic applications, as the knowledge of the present species at a given pH value influences the catalytic activity.^21^ For example, at pH 7, it is evident that a significant amount of the molybdocene complex is in its dimeric form, which could play a significant role during catalysis and has not been previously considered.

### Titration of Aqueous Tungstocene

The two analogous tungstocene complexes [Cp_2_WCl_2_] (**2a**) and [Cp_2_W­(μ-OH)_2_WCp_2_]­(*p*TsO)_2_ (**2b**) were previously described; however, no data in water is available.^33,38^ Thus, the ^1^H NMR spectra were measured in D_2_O (Figures S9 and S11). While with **2a** two Cp signals (at 6.07 and 5.88 ppm) are apparent, with **2b** only one signal appears at 5.93 ppm. This is similar to the analogous molybdocenes (Figures S4 and S6), where **1a** shows two Cp signals (at 6.08 and 5.89) and **1b** shows one dominant signal at 5.95 ppm. The occurrence of two signals for **2a**, **1a,** and **1b** suggests that both mono- and dimeric species are present. Furthermore, dissolving **1a** and **1b** as well as **2a** and **2b**, respectively, at the same pH value (in buffered solutions) reveals the same Cp shifts for mono- and dimeric species (Figures S16–S19). For this, a 0.5 M MOPS solution in D_2_O was prepared and adjusted with 2 M NaOD solution to a pD of 6.55. This is equivalent to a pH of 7 using a correction factor of 0.45.^40^

In the absence of speciation data and for comparative reasons, we performed basic titrations, identical to those for the molybdocenes. Again, aqueous solutions were acidified to a pH value of 2 and titrated with 0.01 M NaOH until a pH of around 11. The resulting titration curves are shown in Figure 3.

**Figure 3:**
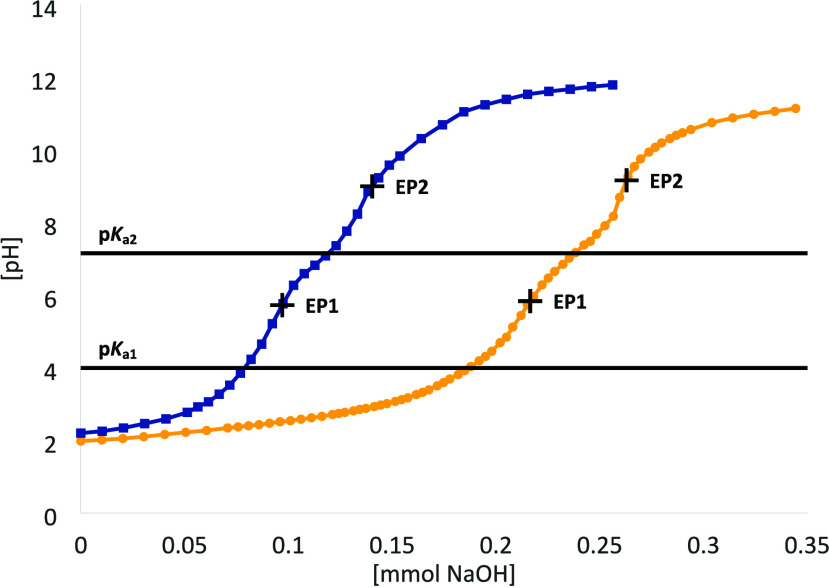
Titration curve for an aqueous solution of **2a** (yellow circles) or **2b** (blue squares). The shown data points represent each measurement point. Solutions of **2a** and **2b** were acidified to a pH of 2 and then titrated with a 0.01 M NaOH solution. The calculated p*K*_a_ values are given as bold black lines. EPs are given as black + symbols.

Titrations of the tungstocene complexes reveal several differences compared to their molybdenum analogues: (a) Only two EPs were observed. (b) Equal amounts of base equivalents were needed per metal center for both compounds (see Table 1). (c) p*K*_a_ values are more acidic compared to that of the molybdenum analogues (Table 2). This suggests that both complexes form the monomeric species predominantly (Scheme 2) and that the [Cp_2_W]^2+^ system is more acidic than the [Cp_2_Mo]^2+^ system.

**Scheme 2:**
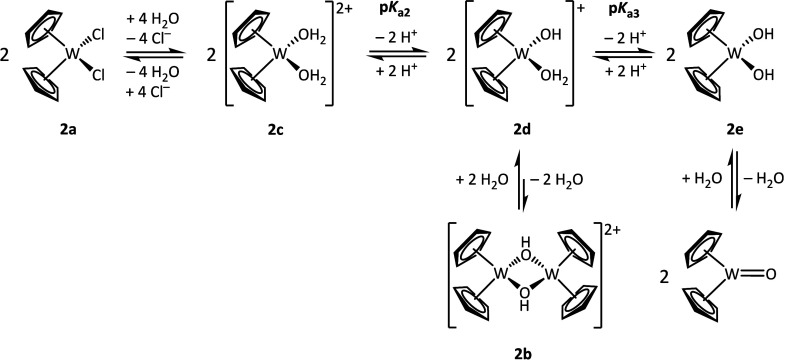
Suggested Acid–Base Equilibria for [Cp_2_W]^2+^

This is similar to the easier comproportionation of Mo­(IV) monoxido and Mo­(VI) dioxido compounds forming Mo­(V) μ-oxido dimers, which is much rarer for tungsten analogues.^41,42^ The observed acidity of the higher homologue W is similar to the higher acidity of tungstic acid compared to molybdic acid.^43,44^ Generally, any water coordinated on a tungsten center is significantly more acidic than that on its molybdenum counterpart. This information is of relevance not only generally for their reactivity in aqueous solutions^22^ but also for explaining reactivity differences of molybdoenzymes compared to tungstoenzymes as they often contain coordinated water molecules in their catalytic cycle.^6^ While differences in reactivity are often explained by the different redox potentials metals,^10^ here, we show that the p*K*_a_ values may be equally used to differentiate their reactivity. Furthermore, the p*K*_a_ values may be used as a measure of their Lewis acidity, suggesting that, here, W appears to be more Lewis acidic than Mo.

### Tosylate Complexes

The precursor complexes **1a**, **1b**, **2a,** and **2b** exhibit differences not only in the species they form upon hydrolysis but also in their counterions. **1a** and **2a** yield Cl^–^ ions, whereas **1b** and **2b** contain *p*TsO^–^ ions due to their synthesis via the tosylate complexes [Cp_2_Mo­(*p*TsO)_2_] (**3**) and [Cp_2_W­(*p*TsO)_2_] (**4**). We were curious whether these two tosylate complexes can be directly hydrolyzed to aqua/hydroxy species similar to the dichlorides, as both counterions, Cl^–^ and *p*TsO^–^, are weak bases.

The tosylate complexes **3** and **4** are mentioned in the literature; however, no characterization data are available, either in solution or in the solid state.^38^ Thus, we prepared both of these following literature procedures. They proved to be poorly soluble, so that characterization in solution was possible only in DMF-*d*_7_. Due to the tosylate groups slowly exchanging in the solution, three distinct species could be observed. Therefore, in the ^1^H NMR spectrum of **3** and **4**, three resonances corresponding to Cp rings could be observed: at 6.31 (Mo) and 6.27 (W) ppm for nonsubstituted [Cp_2_M­(*p*TsO)_2_], at 6.36 (Mo) and 6.33 (W) for monosubstituted [Cp_2_M­(*p*TsO)­(DMF-*d*_7_)]^+^, and at 6.42 (Mo and W) ppm for fully substituted [Cp_2_M­(DMF-*d*_7_)_2_]^2+^. The solid-state structures were determined by a single-crystal X-ray diffraction analysis. Suitable single crystals were obtained directly from the reaction mixture during preparation in acetone. Crystallographic data are given in the SI (Tables S6–S11). Molecular views are displayed in Figure 4.

**Figure 4:**
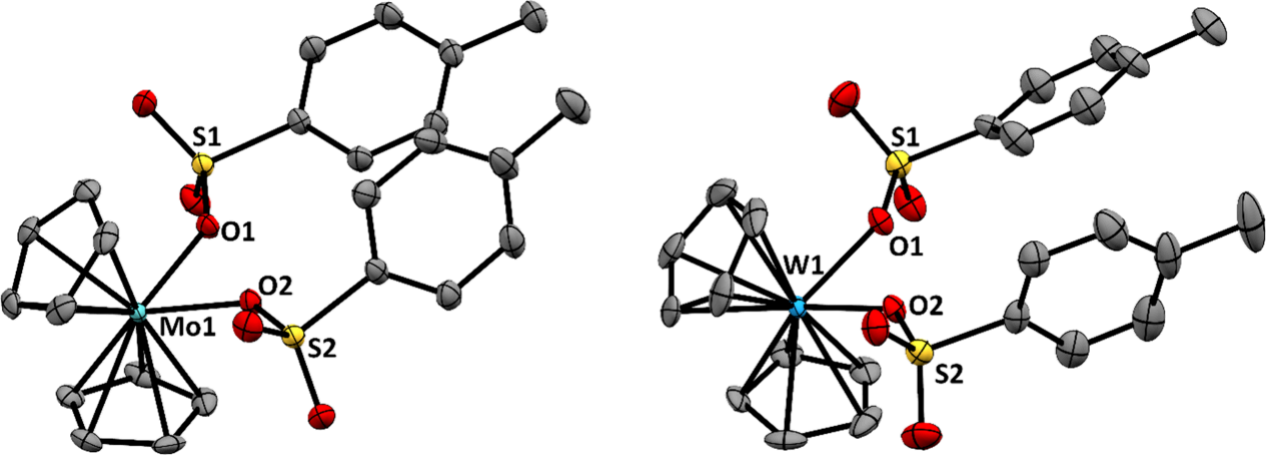
Molecular views of [Cp_2_Mo­(*p*TsO)_2_] (**3**, left) and [Cp_2_W­(*p*TsO)_2_] (**4**, right). The probability ellipsoids are drawn at the 50% level. H atoms were omitted for the sake of clarity.

Both **3** and **4** exhibit a typical bent metallocene structure. The metal–carbon bond lengths are similar across the two metals, with the Mo1–C bond lengths ranging from 2.234(2) to 2.3709(19) Å, and the corresponding W1–C bond lengths ranging from 2.235(5) to 2.366(5) Å. The coordinated sulfonate ligands appear to lie in a plane for both metals, with the torsion angle of S1–O1–Mo1–O2 measuring 178.05(15)° and the corresponding angle of S1–O1–W1–O2 measuring 172.6(4)°. The two Cp ligands are positioned above and below these planes. Additionally, the metal–oxygen bond lengths are comparable, with Mo1–O1 measuring 2.1256(13) Å and W1–O1 measuring 2.113(3) Å. These bond lengths are similar to those of previously reported molybdocene [(MeCp)_2_Mo­(*p*TsO)_2_] (Mo1–O1 2.122(3) Å) and tungstocene [Cp_2_W­(trop)]­(*p*TsO) (W1–O1 2.105(5) Å) compounds.^45,46^

In contrast to **1a** and **2a**, the hydrolysis of **3** and **4** proved to be challenging. The progress of dissolution was investigated by adding D_2_O and recording the ^1^H NMR spectra. At room temperature, both tosylate compounds were found to be largely insoluble in water. Stirring the suspensions overnight did not enhance the solubility, unlike the behavior observed for **1a** and **2a**. Increasing the temperature of the aqueous solutions to 80 °C facilitated the dissolution of the solids, leading to the formation of the expected [Cp_2_Mo­(OH_2_)]^2+^ (**1c**) and [Cp_2_W­(OH_2_)]^2+^ (**2c**) ions, as confirmed by ^1^H NMR spectroscopy (6.07 ppm for **1c**; 6.06 ppm for **2c**). However, significant decomposition was also observed, indicated by the appearance of resonances between 2.9 and 4.2 ppm, attributed to the hydrolyzed CpD ligand (Figures S20 and S21). This is particularly evident for the tungstocene, wherein the resulting Cp signal only integrated to 4H–5H versus the two tosylate counterions, suggesting that half of the tungstocene was not hydrolyzed but decomposed.

In contrast, the hydrolysis of **3** showed a higher conversion to the desired species, where an integral of approximately 8H was observed for the two Cp groups instead of the expected 10H. However, free Cp signals in the range of 4.2–2.9 ppm are also visible. Thus, while molybdocene resulted in more successful hydrolysis than tungstocene, it is evident that *p*TsO^–^ is not suitable as a ligand for achieving quantitative hydrolysis in either system.

Consequently, tosylate molybdo- and tungstocenes are not suitable as precursors for aqueous chemistry, mainly due to their poor solubility, which requires harsh conditions for dissolving them and competes with their stability.

## Conclusions

The aqueous acid–base properties of the tungstocene [Cp_2_W]^2+^ motif as well as the molybdocene [Cp_2_Mo]^2+^ motif have been investigated by extensive potentiometric studies.

With the aqueous molybdocene, three protons in the pH range of 2–11 can be titrated. Two of these protons derive from coordinated H_2_O molecules of monomeric compounds [Cp_2_Mo­(H_2_O)_2_]^2+^ and [Cp_2_Mo­(OH)­(H_2_O)]^+^, respectively. The third proton likely corresponds to a bridged μ-OH̅ ligand in the dimer [Cp_2_Mo­(μ-OH)­(μ-H_2_O)­MoCp_2_]^3+^. Our data give further insights into the dimerization process of these molybdocenes.

The two known complexes [Cp_2_MoCl_2_] (**1a**) and [Cp_2_Mo­(μ-OH)_2_MoCp_2_]­(*p*TsO)_2_ (**1b**) were hydrolyzed, where the former leads to an acidic solution (pH = 3–4) under the formation of HCl, in contrast to the latter, which results in pH = 5.5–6.5. The titration of **1b** starting from pH = 2 revealed the use of unequal amounts of the base to reach the EPs (different ΔEPs). These data point toward the formation of the equilibrium between monomeric [Cp_2_Mo­(H_2_O)_2_]^2+^ (**1c**) and dimeric [Cp_2_Mo­(μ-OH)­(μ-H_2_O)­MoCp_2_]^3+^ (**1f**) in an approximate ratio of 2:1 at pH = 2. In contrast, due to the acidic properties of **1a** already upon hydrolysis, the dimerization to **1f** does not occur because the pH remains below p*K*_a2_, which is required for dimer formation. Thus, the species remains monomeric upon acidification to pH = 2. The difference between the behaviors of **1a** and **1b** is not observed upon increasing the pH above p*K*_a2_, as here the monomer–dimer equilibration can occur. Therefore, the respective amounts of base to reach the EPs are equal for the two compounds. Furthermore, the titrations allowed the determination of p*K*_a2_ = 5.51 ± 0.33 and p*K*_a3_ = 8.12 ± 0.19, which are similar to those reported in the literature.^24^

The analogous tungstocenes show a simpler aqueous speciation. The dimeric species [Cp_2_W­(μ-OH)_2_WCp_2_] does not appear to be present under aqueous conditions, as only the monomeric species [Cp_2_W­(OH)_*n*_(H_2_O)_2–*n*_]^(2–*n*)+^ was observed in the titrated pH range of 2–11. This is evident by the fact that no difference between the precursors [Cp_2_WCl_2_] (**2a**) and [Cp_2_W­(μ-OH)_2_WCp_2_]­(pTsO)_2_ (**2b**) is observed. Additionally, the values of p*K*_a2_ = 3.99 ± 0.35 and p*K*_a3_ = 7.15 ± 0.13 of the tungstocenes are more acidic by 1–1.5 p*K*a points compared to the molybdocenes.

The complexes [Cp_2_Mo­(*p*TsO)_2_] (**3**) and [Cp_2_W­(*p*TsO)_2_] (**4**) were synthesized and characterized. Their hydrolysis allows the investigation of the influence of the counterion. However, their hydrolysis to [Cp_2_Mo­(H_2_O)_2_]^2+^ proved to be nonquantitative under the formation of side products. This is in contrast to similar dichlorides **1a** and **2a**, which readily undergo complete hydrolysis. The lower solubility of the tosylate complexes required a higher temperature for hydrolysis, which is likely the cause for the side reactions. It is noteworthy that, with the Mo complex, a higher conversion to the desired aqueous species was observed, suggesting a larger stability of [Cp_2_Mo]^2+^ compared to [Cp_2_W]^2+^.

Our data allow a comparison of aqueous molybdenum­(IV) and tungsten­(IV) complexes. We found that the molybdenum system is more prone to dimerization, leading to a more complex behavior in solution. The high tendency of tungsten toward forming monomers has an influence on the choice of supporting ligands in model complexes, as the steric demand can likely be lower, which influences the reactivity. Furthermore, the p*K*_a_ values for the W complexes were found to be lower compared to Mo, rendering the former a stronger Lewis acid. This is of general interest for the understanding of tungstoenzymes where the proton of a coordinated water molecule is likely more prone to an electrophilic attack at biological substrates compared to molybdoenzymes. This suggests that the observed difference in the behaviors of Mo vs W enzymes is not necessarily due to their often-suggested different redox potentials but may also be due to their different acid–base behaviors. This is particularly relevant in the tungstoenzyme *acetylene hydratase* where formally a nonredox reaction occurs.

## Experimental Section

### General

All experiments were performed under exclusion of air by employing standard Schlenk procedures. Aqueous solutions were degassed either by three freeze–pump–thaw cycles or by bubbling nitrogen through the solution.

### Titration Procedure

The desired amount of complex was weighed out under ambient atmosphere using an analytical scale and transferred into a titrating flask under an inert gas flow. The buret was then filled with degassed 0.01 M solutions of titrant (NaOH or HCl, respectively). The complex was then fully dissolved with 10 mL of degassed water before the pH of the solution was set to 2 for basic titrations and 11 for acidic titrations with degassed 1.2 M HCl or 1 M NaOH solution, respectively. The complexes were then titrated at room temperature until a pH of almost 12 or 2 was achieved.

All titrations were recorded at least 4 times, with the exception of the acidic titrations, as these were used only to confirm the results of the basic titrations.

## Supplementary Material



## Notes

Synthetic procedure of the complexes; crystallographic data of **2a**, **3**, and **4**; ^1^H and ^13^C NMR spectra of the compounds and titration results; also available at Zenodo at 10.5281/zenodo.17037086 (PDF)
