# Supplementary material for: Comparative Study of Aqueous Acid–Base Properties of Tungstocene and Molybdocene Complexes
Source: Organometallics. 2025 Dec 12;45(4):402–9. doi: 10.1021/acs.organomet.5c00345 (PMC12933890; doi:10.1021/acs.organomet.5c00345)
Supplement: Supplementary file 1 [file om5c00345_si_001.pdf]

Supporting Information

Comparative Study of Aqueous Acid-Base Properties of Tungstocene and Molybdocene Complexes

*Niklas Stix, Miljan Z. Ćorović, Sophia S. Schiller, Antoine Dupé, and Nadia C. Mösch-Zanetti**

Institute of Chemistry, Inorganic Chemistry, University of Graz, 8010 Graz, Austria

* Corresponding author. E-mail address: nadia.moesch@uni-graz.at

Table of Contents

1 General Considerations 3

2 Syntheses 4

3 Titration Results 11

4 NMR Spectra 14

5 Crystal Structure Determination 26

5.1. [Cp_2WCl_2] (2a) 28

5.2. [$Cp_2Mo(p$TsO$)_2$] (3) 30

5.3. [$Cp_2W(p$ToS$)_2$] (4) 32

6 References 34

1 General Considerations

All experiments were performed using standard Schlenk techniques unless stated otherwise. Commercially available chemicals were used as received except for $MoCl_5$ and WCl_6. These were purified by sublimation at 130 °C under a pressure of less than 0.15 mbar. Air and moisture-sensitive chemicals were stored in a Schlenk flask or under N_2 atmosphere in a glovebox. NaOD solutions were prepared by dissolving metallic sodium in D_2O. Dioxygen-free H_2O and D_2O were obtained by flushing with N_2 for 15min. The NaOH, HCl and MOPS-buffer solutions used for titrations and NMR spectroscopy were purged of oxygen by performing three freeze-pump-thaw cycles. All other solvents were purified by a Pure Solv Solvent Purification System and stored over activated molecular sieves (3 or 4 Å). NaCp was prepared according to published procedures.[1] NMR spectra were recorded using a Bruker Avance III spectrometer. ^{1}H NMR spectra were recorded at 300 MHz and referenced to the residual protons of the NMR solvents. ^{13}C NMR spectra were obtained at 75 MHz, and spectra were referenced to the deuterated solvent peak. The chemical shifts δ are given in ppm. The multiplicity of peaks is denoted as singlet (s), doublet (d), triplet (t), quadruplet (q) or multiplet (m). Coupling constants J are given in Hertz. IR spectra were recorded in the solid-state at a resolution of 2 cm^{-1} on a Bruker Alpha-P Diamond ATR-FTIR or an Anton Paar Lyza 7000 at a resolution of 1.4 cm^{-1}. Elemental analyses (C, H, S) were carried out by the Department of Inorganic Chemistry at the Graz University of Technology (Heraeus Vario Elementar automatic analyser). pH measurements were recorded with the XS Instruments pH 70+DHS® or a Mettler Toledo Seven2Go Pro S8 using an inLab Science Pro-ISM Electrode.

2 Syntheses

[Cp_2MoH_2]. Modified procedure from literature.[2]

A flask was charged with $MoCl_5$ (1.470g; 1.00 equiv), $NaBH_4$ (0.519 g; 2.55 equiv) and NaCp (3.869 g; 8.16 equiv). Then, 30 mL of pentane was added to suspend the solids. The apparatus was cooled to -80 °C. A dropping funnel was charged with 40 mL of THF, and the apparatus was closed with a bubbler. The THF was added dropwise over about 1h. When all the THF had been added, the mixture was allowed to warm to room temperature before exchanging the dropping funnel for a reflux condenser. The mixture was then stirred under reflux for 16 h. Afterwards, the mixture was allowed to cool to room temperature under a flow of N_2, before removing all volatiles *in vacuo*. The remaining solids were extracted with 3 x 25 mL of Et_2O. The extracts were dried *in vacuo* to yield the crude product as a red solid. The pure product was isolated via sublimation at < 0.3 mbar and 100 °C. This yielded the product as yellow crystals (yield 0.283 g, 23%).

^1H NMR (300 MHz, C_6D_6) δ 4.36 (s, 10H, Cp), -8.78 (s, 2H, Mo-H) ppm.

[Cp_2WH_2]. Modified procedure from literature.[2]

A flask was charged with WCl_6 (1.000g; 1.00 equiv), $NaBH_4$ (0.238 g; 2.50 equiv) and NaCp (1.333 g; 6.00 equiv). Then, 15 mL of pentane was added to suspend the solids. The apparatus was cooled to -80 °C. A dropping funnel was charged with 20 mL of THF, and the apparatus was closed with a bubbler. The THF was added dropwise over about 1h. When all the THF had been added, the mixture was allowed to warm to room temperature before exchanging the dropping funnel for a reflux condenser. The mixture was then stirred under reflux for 20 h. Afterwards, the mixture was allowed to cool to room temperature under a flow of N_2, before removing all volatiles *in vacuo*. The remaining solids were extracted with 3 x 10 mL of Et_2O. The extract was then dried *in*

vacuo to yield the crude product as a red solid. The pure product was then isolated via sublimation at < 0.3 mbar and 100 °C. This yielded the product as yellow crystals (yield 0.150 g, 19%).

^{1}H NMR (300 MHz, C_6D_6) δ 4.24 (s, 10H, Cp), -12.09 – -12.39 (m, 2H, W-H) ppm.

[Cp_2MoCl_2] (1a). Modified procedure from literature.[3]

[Cp_2MoH_2] (0.183 g; 0.80 mmol) was dissolved in 5 mL of dry $CHCl_3$. This initially formed a yellow solution, which was allowed to sit at room temperature without stirring for 60h. The solution was now green, and dark green crystals had formed, which were isolated and washed with 3x2 mL of pentane before drying *in vacuo*. This gave a crystalline product suitable for single-crystal X-ray diffraction analysis (205 mg; yield 86 %).

^{1}H NMR (300 MHz, $CDCl_3$) δ 5.62 (s, 10H, Cp) ppm.

^{13}C NMR (75 MHz, $CDCl_3$) δ 101.61 (Cp) ppm.

Elemental analysis calculated for $C_{10}H_{10}MoCl_2$: C, 40.44; H, 3.39. Found: C, 40.45; H, 3.18.

[$Cp_2Mo(\mu\text{-}OH)_2MoCp_2$](*p*TsO)$_2$ (1b).

A flask was charged with [Cp_2MoH_2] (0.090 g; 0.44 mmol; 1 equiv) and *p*TsOH • H_2O (0.075 g; 0.44 mmol; 1 equiv). This was dissolved in 10 mL of acetone, followed by the addition of 100 µL of degassed H_2O. The reaction mixture was then stirred under reflux for 16h, giving the product as a grey-green precipitate. Afterwards, the reaction mixture was cooled to 0 °C before isolation via filtration. The product was extracted with 3 x 5

mL of MeOH into a new flask. After removing the solvent *in vacuo*, the product was obtained as flat, dark-green crystals (0.125 g; 76% yield).

^{1}H NMR (300 MHz, CD_3OD) δ 7.69 (d, *J* = 8.2 Hz, 4H, *p*TsO-*Ar*-H), 7.24 (d, *J* = 7.9 Hz, 4H, *p*TsO-*Ar*-H), 6.13 (s, Cp), 6.01 (s, Cp), 5.86 (s, Cp), 2.37 (s, 6H, *p*TsO-*Me*-H) ppm.

^{13}C NMR (75 MHz, CD_3OD) δ 141.80 (*Ar*-C), 129.88 (*Ar*-C), 126.96 (*Ar*-C), 103.99 (Cp), 103.56 (Cp), 21.30 (*Me*-C) ppm.

Elemental analysis calculated for $C_{34}H_{36}O_6S_2Mo_2$: C, 49.28; H, 4.38; S, 7.74. Found: C, 48.95; H, 4.41; S, 7.37.

[Cp_2WCl_2] (2a). Modified procedure from literature.[3]

[Cp_2WH_2] (0.100 g; 0.316 mmol) was dissolved in 5 mL of dry $CHCl_3$. This initially formed a yellow solution, which was allowed to sit at room temperature without stirring for 48h. Afterwards, the solution was green, and dark green crystals had formed, which were isolated and washed with 3x2 mL of pentane before drying *in vacuo*. This gave a crystalline product suitable for single-crystal X-ray diffraction analysis (110 mg; yield 90 %).

^{1}H NMR (300 MHz, $CDCl_3$) δ 5.60 (s, 10H, Cp).

^{13}C NMR (75 MHz, $CDCl_3$) δ 96.84 ppm.

Elemental analysis calculated for $C_{10}H_{10}WCl_2$: C, 31.20; H, 2.62. Found: C, 31.20; H, 2.36.

[$Cp_2W(\mu\text{-}OH)_2WCp_2$](*p*TsO)$_2$ (2b). Modified procedure from literature.[4]

A flask was charged with [Cp_2WH_2] (0.500 g; 0.95 mmol; 1 equiv) and *p*TsOH • H_2O (0.301 g; 0.95 mmol; 1 equiv). This was dissolved in 50 mL of acetone, followed by the addition of 500 µL of degassed H_2O. The reaction mixture was then refluxed for 16h, yielding the crude product as a grey precipitate. Afterwards, the reaction mixture was cooled to 0 °C before isolation via filtration. The product was extracted with 3 x 20 mL of MeOH into a new flask. The solvent was then removed *in vacuo*. Lastly, the product was washed with 4 x 15 mL of Et_2O. After drying *in vacuo*, the product was obtained as a grey-green powder (0.636 g; 80% yield).

Crystals suitable for X-ray crystal structure analysis were obtained by dissolving in MeOH and slow evaporation at -36 °C over two weeks.

^{1}H NMR (300 MHz, CD_3OD) δ 7.70 (d, *J* = 8.2 Hz, 4H, *p*TsO-*Ar*-H), 7.25 (d, *J* = 7.9 Hz, 4H, *p*TsO-*Ar*-H), 6.16 (s, Cp), 5.76 (s, Cp), 2.38 (s, 6H, *p*TsO-*Me*-H) ppm.

^{13}C NMR (75 MHz, CD_3OD) δ 141.77 (*p*TsO-*Ar*-C), 129.87 (*p*TsO-*Ar*-C), 126.99 (*p*TsO-*Ar*-C), 98.93 (Cp), 98.37 (Cp), 21.31 (*p*TsO-*Me*-C) ppm.

Elemental analysis calculated for $C_{34}H_{36}O_6S_2W_2 \cdot 1.1\ H_2O$: C, 39.87; H, 3.76; S, 6.26. Found: C, 40.17; H, 3.65; S, 5.96.

[Cp_2Mo(*p*TsO)$_2$] (3). Modified procedure from literature.[4]

A flask was charged with [Cp_2MoH_2] (0.078 g; 0.247 mmol; 1 equiv) and water-free *p*TsOH (0.085 g; 0.494 mmol; 2 equiv). To this, 3 mL of acetone were added. This synthesis proceeded much quicker than observed for the corresponding tungsten compound (**4**), and a green precipitate formed immediately. Therefore, the mixture, which was already starting to form product, was filtered over celite immediately. Green crystals suitable for X-ray analysis could then be obtained from letting the filtrate rest overnight.

[Cp_2Mo(*p*TsO)$_2$]

^{1}H NMR (300 MHz, DMF-d_7) δ 7.52 (d, J = 8.1 Hz, 4H, *p*TsO-*Ar*-H), 7.23 (d, J = 7.9 Hz, 4H, *p*TsO-*Ar*-H), 6.31 (s, 10H, Cp-H), 2,36 (s, 6H, pTsO-*Me*-H) ppm.

^{13}C NMR (75 MHz, DMF-d_7) δ 130.03 (*p*TsO-*Ar*-C), 126.88 (*p*TsO-*Ar*-C), 104.78 (Cp-C), 21.46 (pTsO-*Me*-C) ppm.

[Cp_2Mo(*p*TsO)(DMF-d_7)]$^+$

^{1}H NMR (300 MHz, DMF-d_7) δ 7.65 (d, J = 7.9 Hz, 2H, *p*TsO-*Ar*-H), 7.33 (d, J = 7.8 Hz, 2H, *p*TsO-*Ar*-H), 6.36 (s, 10H, Cp-H), 2,38 (s, 3H, *p*TsO-*Me*-H) ppm.

^{13}C NMR (75 MHz, DMF-d_7) δ 130.29 (*p*TsO-*Ar*-C), 126.88 (*p*TsO-*Ar*-C), 105.72 (Cp-C), 21.46 (*p*TsO-*Me*-C) ppm.

[Cp_2Mo(DMF-d_7)$_2$]$^{2+}$

^{1}H NMR (300 MHz, DMF-d_7) δ 6.42 (10H, Cp-H) ppm.

^{13}C NMR (75 MHz, DMF-d_7) δ 106.57 (Cp-C) ppm.

(pTsO)$^-$

^{1}H NMR (300 MHz, DMF-d_7) δ 7.63 (d, J = 8.12 Hz, 2H, *Ar*-H) 7.12 (d, J = 7.7 Hz, 2H, *Ar*-H) 2.30 (s, 3H, *Me*-H) ppm.

^{13}C NMR (75 MHz, DMF-d_7) δ 129.02 (*Ar*-C), 126.95 (*Ar*-C), 21.46 (*Ar*-C) ppm.

Note: A mixture of species is present as the tosylate is slowly exchanged by DMF-d_7.

Elemental analysis calculated for $C_{24}H_{24}MoO_6S_2 \cdot 0.06\ C_3H_6O$: C, 50.77; H, 4.29; S, 11.21. Found: C, 50.87; H, 3.95; S, 10,87.

[$Cp_2W(p$TsO)$_2$] (4). Modified procedure from literature.[4]

A solution of [Cp_2WH_2] (0.100 g; 0.316 mmol; 1 equiv) in 2 mL of acetone and a solution of *p*TsOH (0.109 g; 0.633 mmol; 2 equiv) in 1.5 mL of acetone were prepared. These were then combined in a flask without stirring. After sitting for 18h, dark-violet, needle-like crystals had formed. These were isolated by reducing the volume *in vacuo* to about 0.5 mL, followed by filtration. The product was then washed with 2 x 2 mL of Et_2O before drying *in vacuo*. This gave the product as crystals suitable for single-crystal X-ray diffraction analysis (yield 0.126 g; 61 %).

[$Cp_2W(p$TsO)$_2$]

^{1}H NMR (300 MHz, DMF-d_7) δ 7.54 (d, J = 8.3 Hz, 4H, *p*TsO-*Ar*-H), 7.25 (d, J = 8.0 Hz, 4H, *p*TsO-*Ar*-H), 6.27 (s, 10H, Cp-H), 2.36 (s, 6H, *p*TsO-*Me*-H) ppm.

^{13}C NMR (75 MHz, DMF-d_7) δ 130.11 (*p*TsO-*Ar*-C), 126.97 (*p*TsO-*Ar*-C), 99.21 (Cp-C), 21.47 (pTsO-*Me*-C) ppm.

[$Cp_2W(p$TsO)(DMF-d_7)]$^+$

^{1}H NMR (300 MHz, DMF-d_7) δ 7.67 (d, J = 8.2 Hz, 2H, *p*TsO-*Ar*-H), 7.35 (d, J = 7.8 Hz, 2H, *p*TsO-*Ar*-H), 6.33 (s, 10H, Cp-H), 2,38 (s, 3H, *p*TsO-*Me*-H) ppm.

^{13}C NMR (75 MHz, DMF-d_7) δ 130.34 (*p*TsO-*Ar*-C), 127.01 (*p*TsO-*Ar*-C), 100.08 (Cp-C), 21.47 (*p*TsO-*Me*-C) ppm.

$[Cp_2W(DMF\text{-}d_7)_2]^{2+}$

^{1}H NMR (300 MHz, DMF-d_7) δ 6.42 (10H, Cp-H) ppm.

^{13}C NMR (75 MHz, DMF-d_7) δ 100.91 (Cp-C) ppm.

(*p*TsO)$^{-}$

^{1}H NMR (300 MHz, DMF-d_7) δ 7.66 (d, J = 8.12 Hz, 2H,*Ar*-H) 7.12 (d, J = 7.7 Hz, 2H, *Ar*-H) 2.30 (s, 3H, *Me*-H) ppm.

^{13}C NMR (75 MHz, DMF-d_7) δ 129.07 (*Ar*-C), 126.94 (*Ar*-C), 21.47 (*Me*-C) ppm.

Note: A mixture of species is present as the tosylate is slowly exchanged by DMF-d_7.

Elemental analysis calculated for $C_{24}H_{24}WO_6S_2$: C, 43.92; H, 3.69; S, 9.77. Found: C, 43.65; H, 3.59; S, 9,82.

3 Titration Results

Calculation of pK_a Values. The titration curves were recorded and the plots analyzed using literature described methods.[5] With the help of the 1st and 2nd derivative of the titration curves, the maximum slope of the curve was determined, which corresponds to the equivalence points. These can then be used to determine the half equivalence points ([HA] = [A⁻]). According to the Hendersson Hasselbalch Equation the half equivalence point then corresponds to the pK_a:

$$pH = pK_a + log_{10} \frac{[A^-]}{[HA]}$$

$$pH = pK_a + log_{10} 1$$

$$pH = pK_a$$

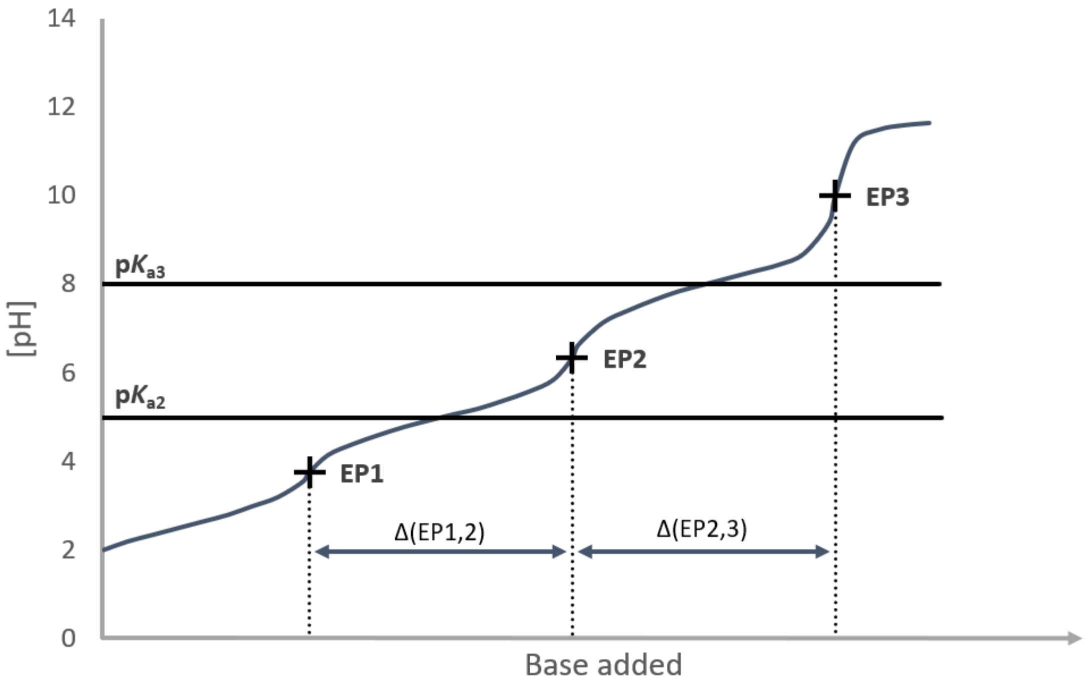

Figure S1: Simulated ideal titration curve where Δ(EP1,2) = Δ(EP1,2) = 1 equivalent of base per complex, to aid in visualization.

Table S1. Obtained pK_a values from each titration.

Basic titrations		
**#**	**pK_{a2}**	**pK_{a3}**
**2b**		
1	3.77	7.13
2	3.54	7.07
3	3.88	7.11
4	3.89	6.95
Arithmetic mean of 4 measurements ± SD	3.77 ± 0.16	7.06 ±0.08
**2a**		
1	4.54	7.24
2	4.38	7.31
3	3.94	7.25
4	4.07	7.35
Mean ± SD	4.23 ± 0.27	7.29 ± 0.05
**1b**		
1	5.84	8.21
2	5.68	8.29
3	5.62	8.11
4	5.98	8.18
Mean ± SD	5.78 ± 0.16	8.20 ± 0.07
**1a**		
1	5.56	8.25
2	5.54	8.24
3	5.47	8.21
4	5.64	8.20
Mean ± SD	5.55 ± 0.07	8.23 ± 0.02

Acidic titrations		
**#**	**pK_{a2}**	**pK_{a3}**
**1b**	4.99	7.69
**1a**	4.84	7.81

Table S2. Obtained titrant equivalents for Δ(EP1,2) and Δ(EP2,3).

Basic titrations		
**#**	**Δ(EP1,2)**	**Δ(EP2,3)**
**2b**		
1	2.01	
2	1.97	
3	2.05	
4	2.42	
Mean ± SD	2.11 ± 0.21	
**2a**		
1	1.15	
2	1.01	
3	0.85	
4	1,01	
Mean ± SD	1.01 ± 0.120	
**1b**		
1	1.41	1.66
2	1.14	1.76
3	1.11	1.67
4	0.82	1.53
Mean ± SD	1.12 ± 0.24	1.65 ± 0.09
**1a**		
1	0.98	0.89
2	1.09	0.97
3	0.98	1.01
4	0.99	0.69
Mean ± SD	1.01 ± 0.05	0.89 ± 0.15
**Acidic titrations**		
**#**	**Δ(EP1,2)**	**Δ(EP2,3)**
**1a**	0.57	0.96
**1b**	0.50	1.00

4 NMR Spectra

[Cp_2MoH_2]

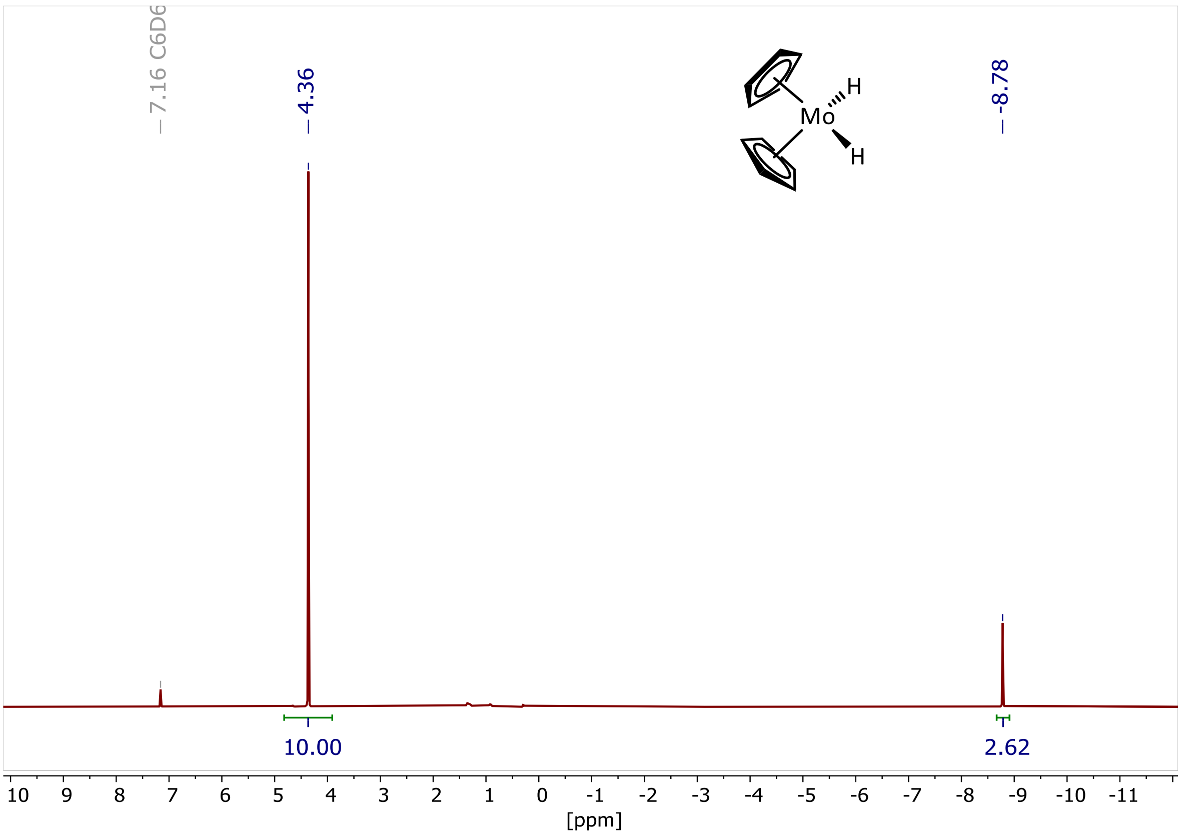

Figure S2: ^{1}H NMR spectrum of [Cp_2MoH_2] in C_6D_6.

[Cp_2MoCl_2] (1a)

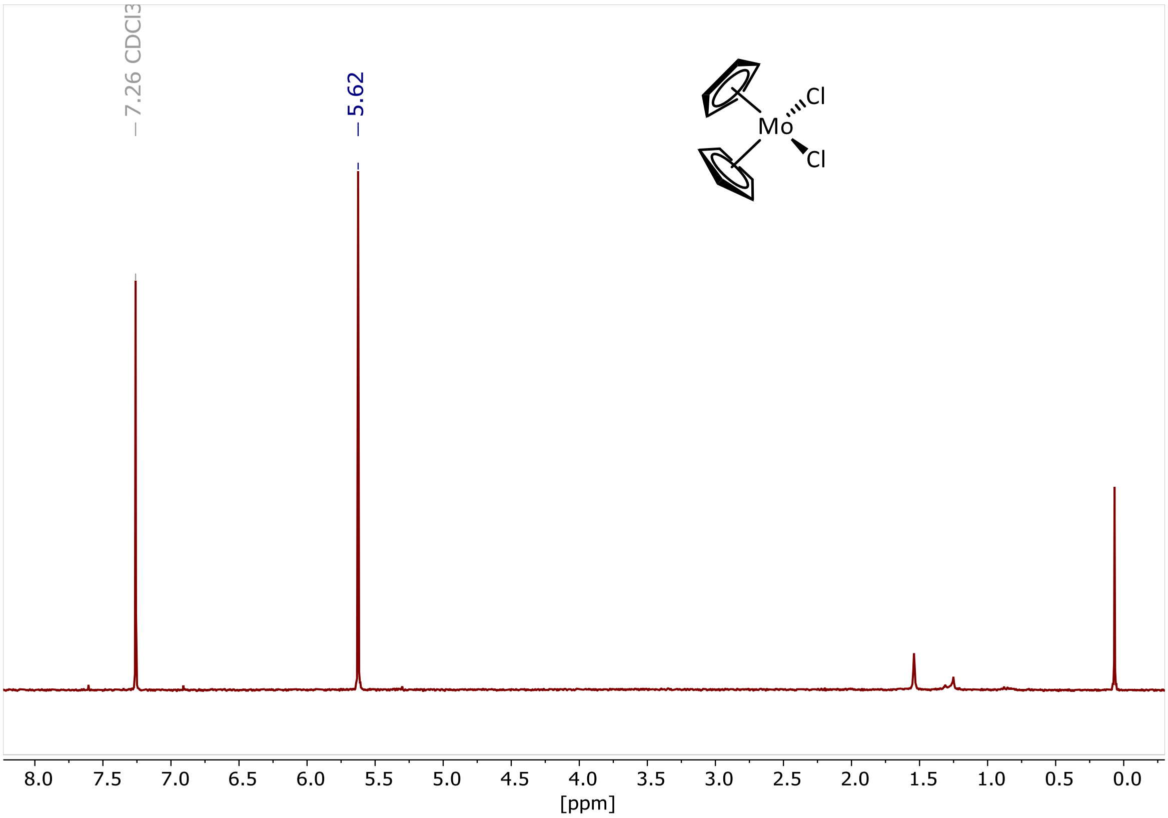

Figure S3: ^{1}H NMR spectrum of **1a** in $CDCl_3$.

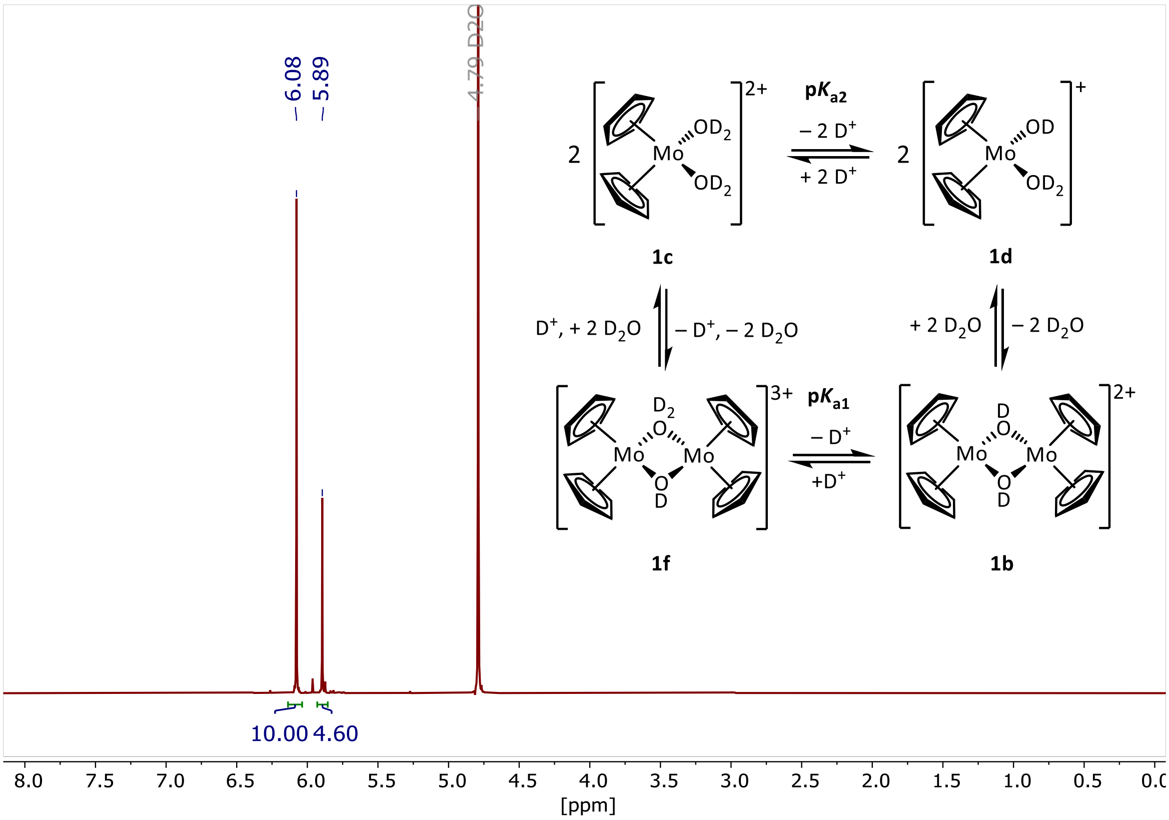

Figure S4: ^{1}H NMR spectrum upon hydrolysis of **1a** in D_2O. Equivalent solutions in H_2O resulted in a pH of about 3.7. Equilibria between **1c** and **1d** as well as their corresponding dimers **1f** and **1b** are found in solution.

[Cp2Mo(μ-OH)2MoCp2](pTsO)2 (1b)

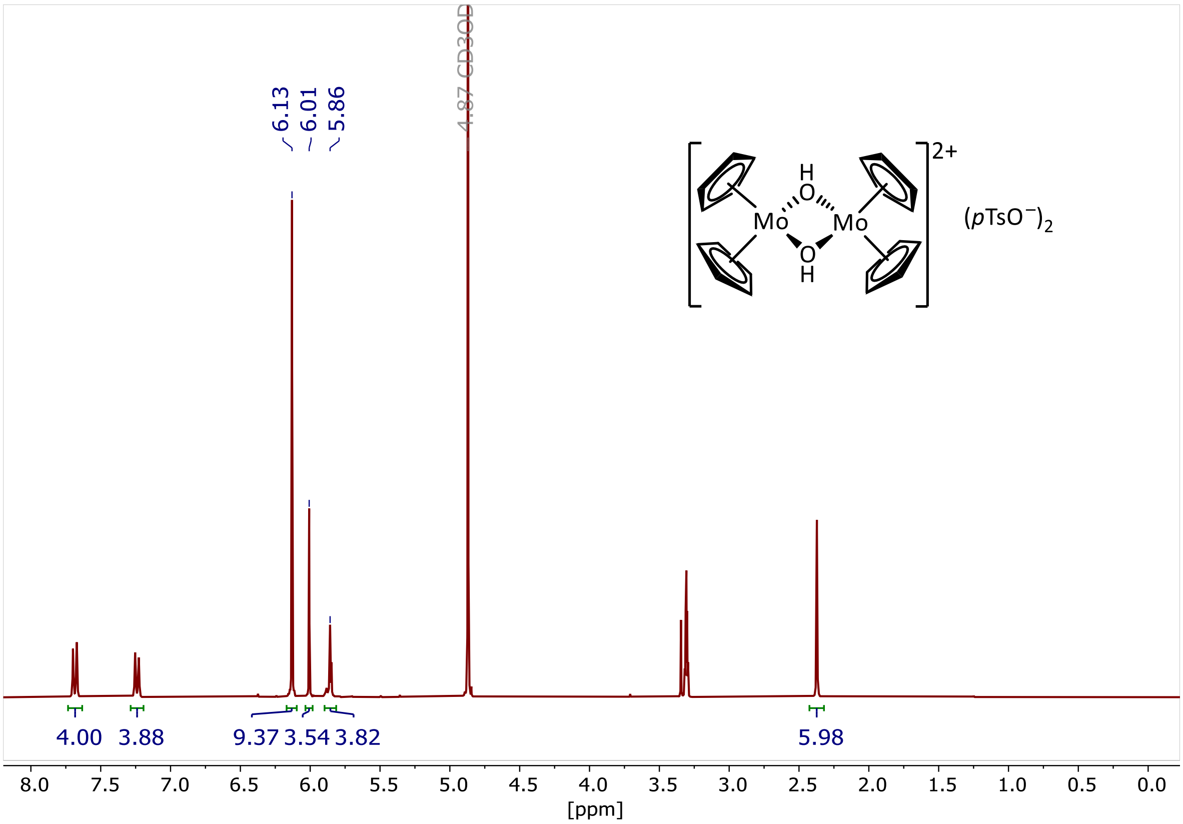

Figure S5: ^{1}H NMR spectrum of **1b** in CD_3OD.

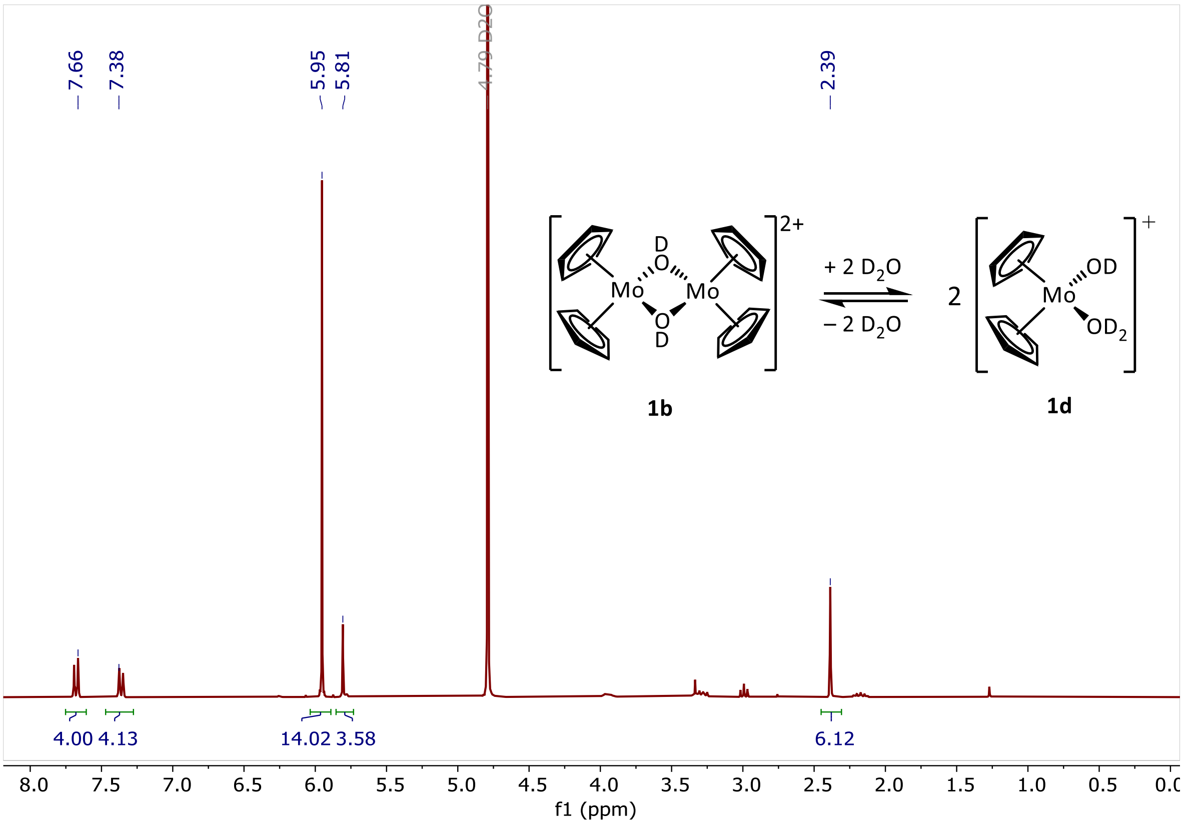

Figure S6: ^{1}H NMR spectrum after hydrolysis of **1b** in D_2O. Equivalent solutions in H_2O resulted in a pH of about 5.8. The two species **1b** and **1d** are in solution.

[Cp_2WH_2]

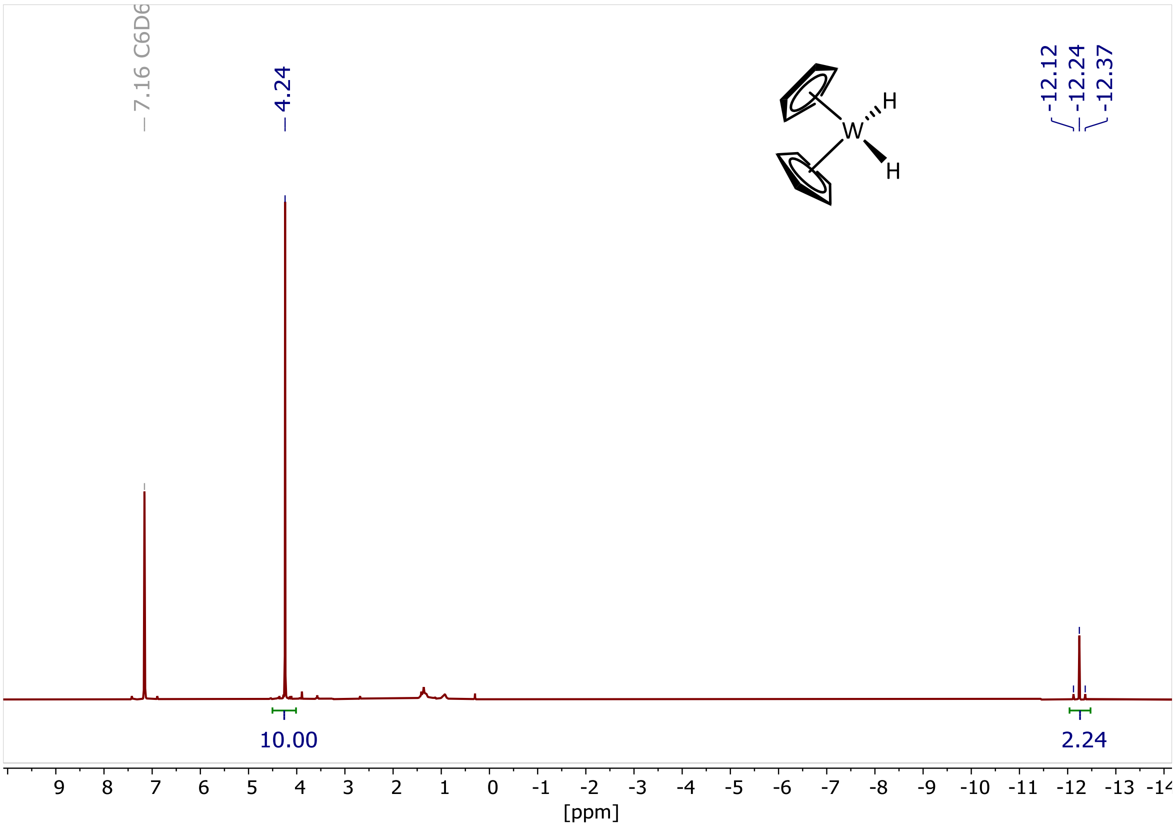

Figure S7: ^1H NMR spectrum of [Cp_2WH_2] in C_6D_6.

[Cp_2WCl_2] (2a)

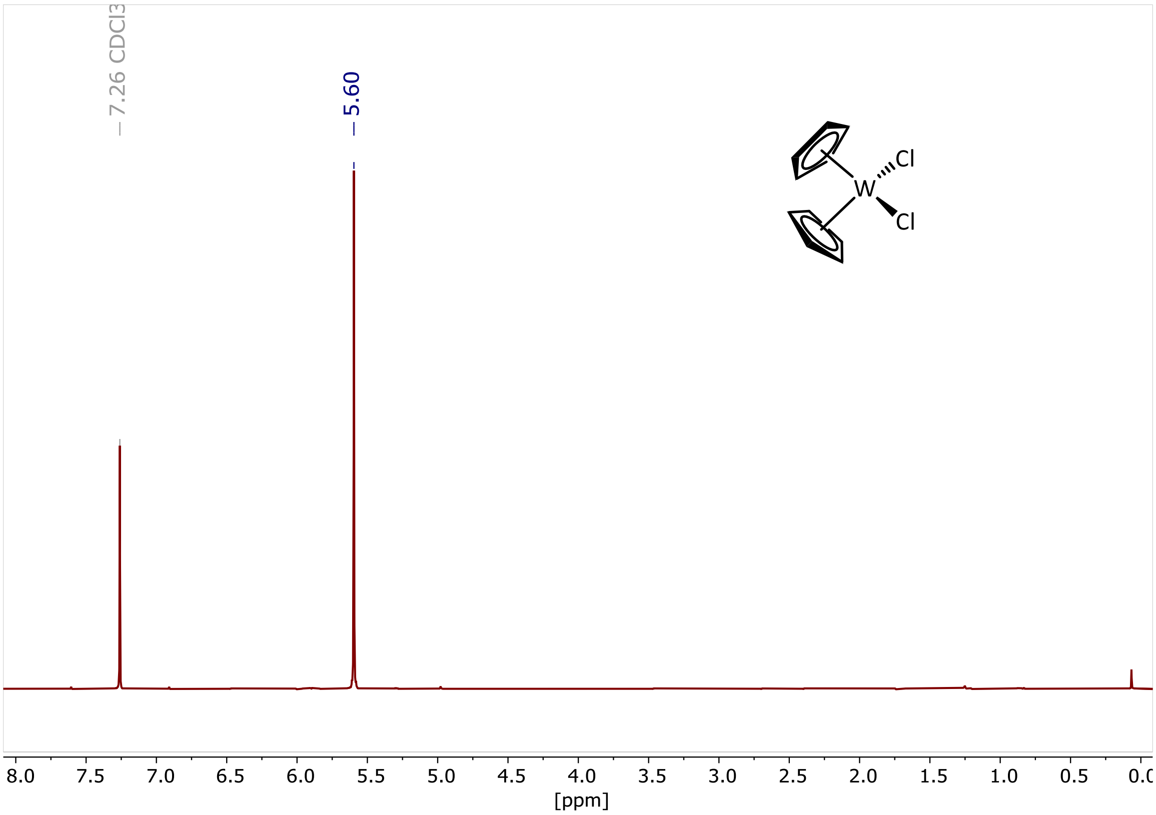

Figure S8: ^{1}H NMR spectrum of **2a** in $CDCl_3$.

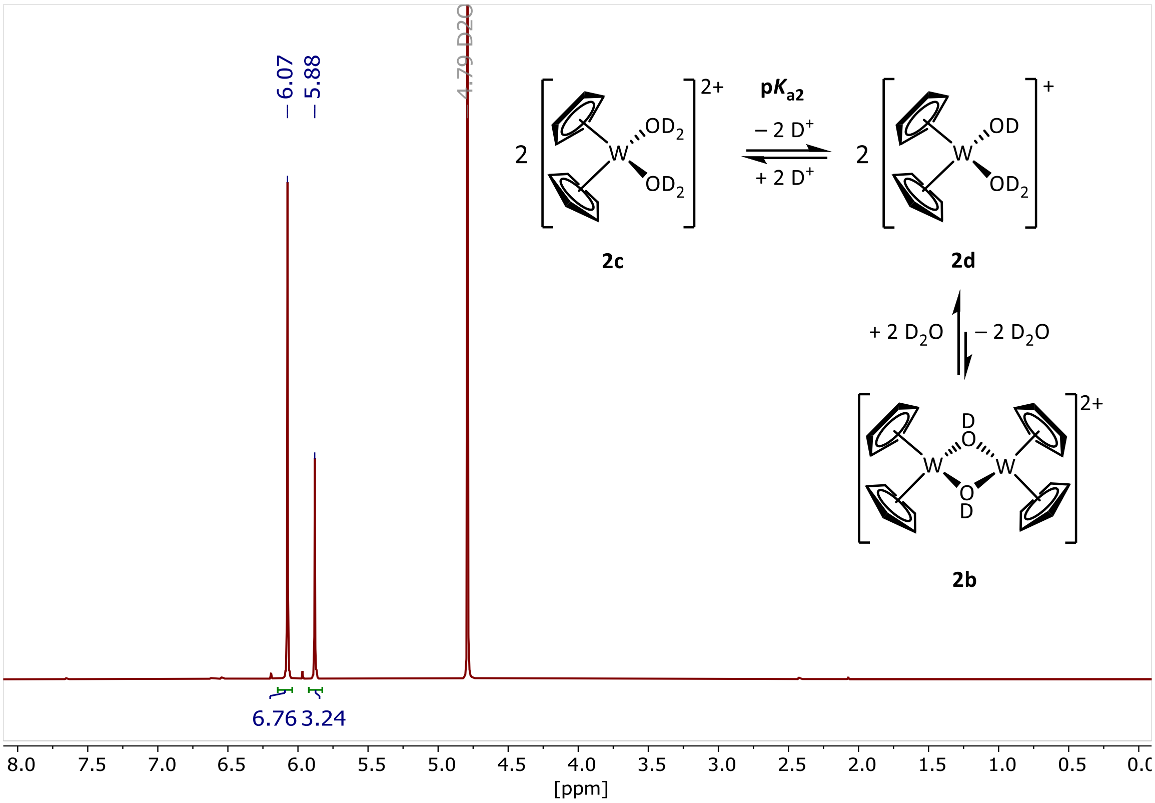

Figure S9. ^{1}H NMR spectrum upon hydrolysis of **2a** in D_2O. Equivalent solutions in H_2O resulted in a pH of about 2.8. An equilibrium between **2c** and **2d** as well as **2b** is found in solution.

[Cp2W(μ-OH)2WCp2](*p*TsO)2 (2b)

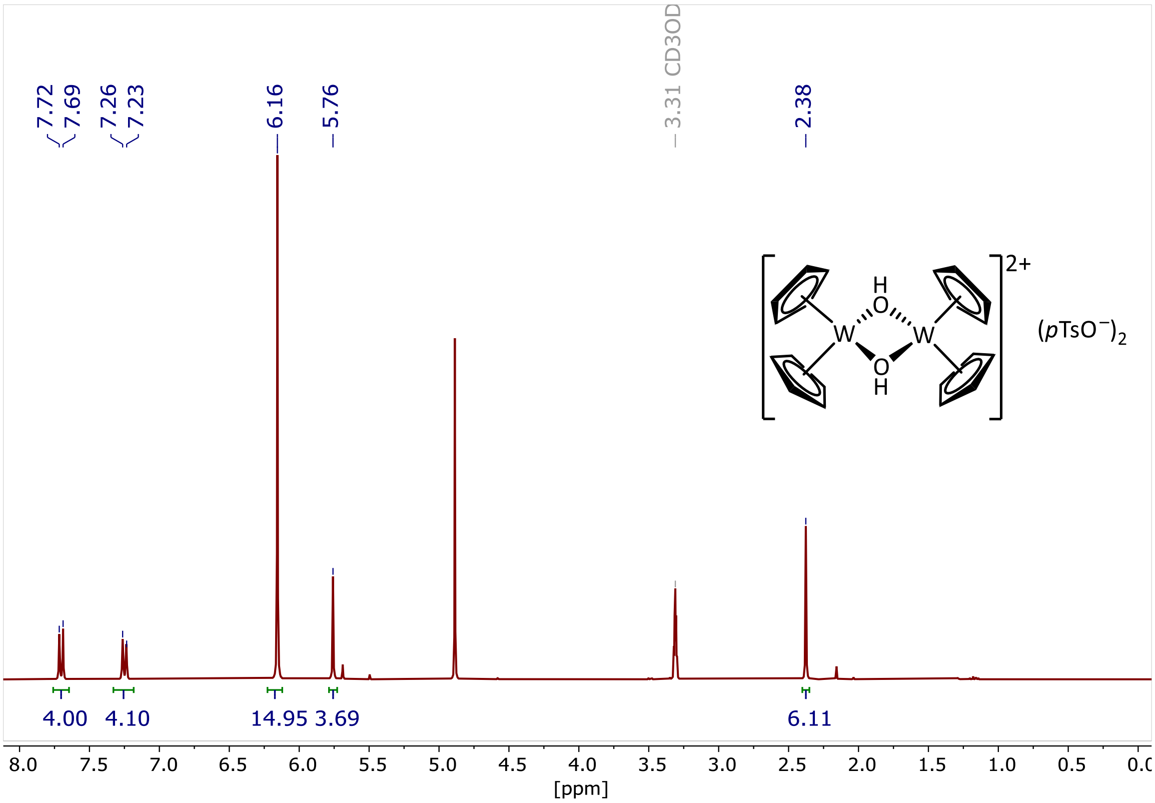

Figure S10. ^{1}H NMR spectrum of **2b** in CD_3OD

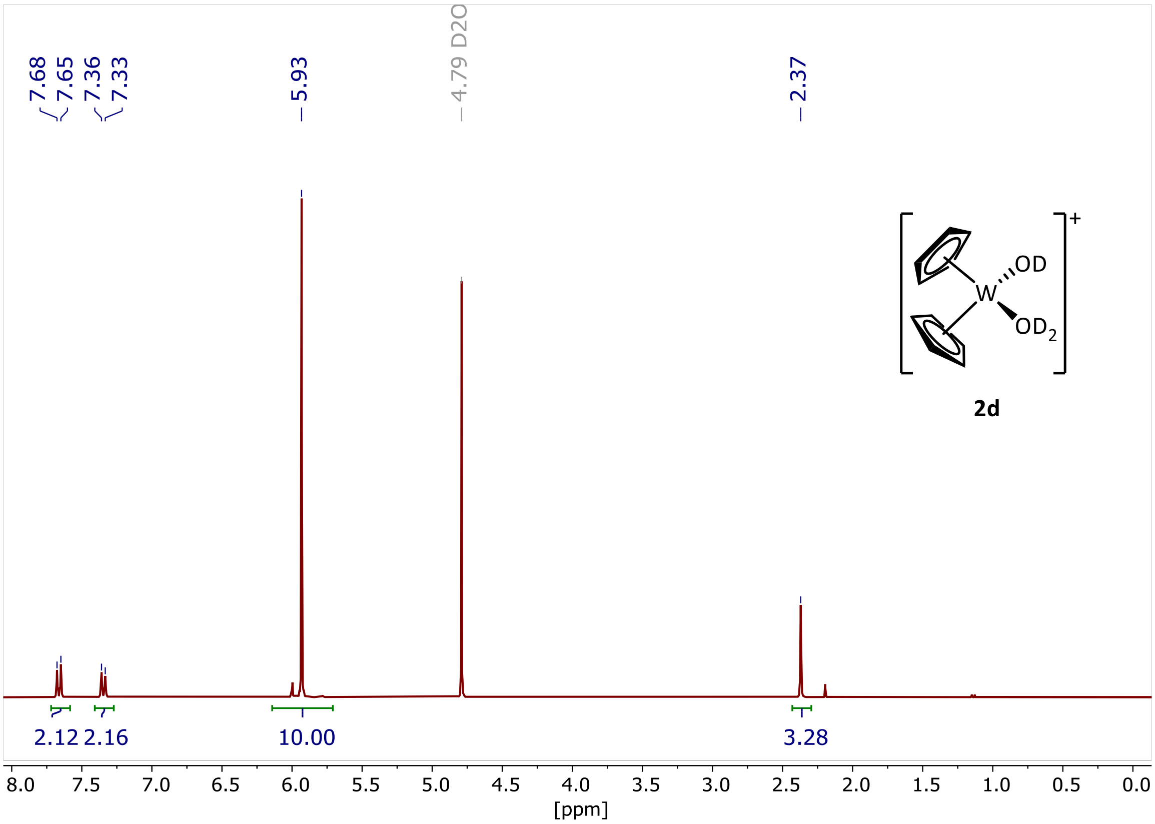

Figure S11. ^{1}H NMR spectrum of **2b** upon hydrolysis in D_2O leading mainly to formation of **2d**. Equivalent solutions in H_2O resulted in a pH of about 4.2. The equilibrium must therefore be towards **2d** in solution.

[$Cp_2Mo(p$TsO$)_2$] (3) and [$Cp_2W(p$TsO$)_2$] (4)

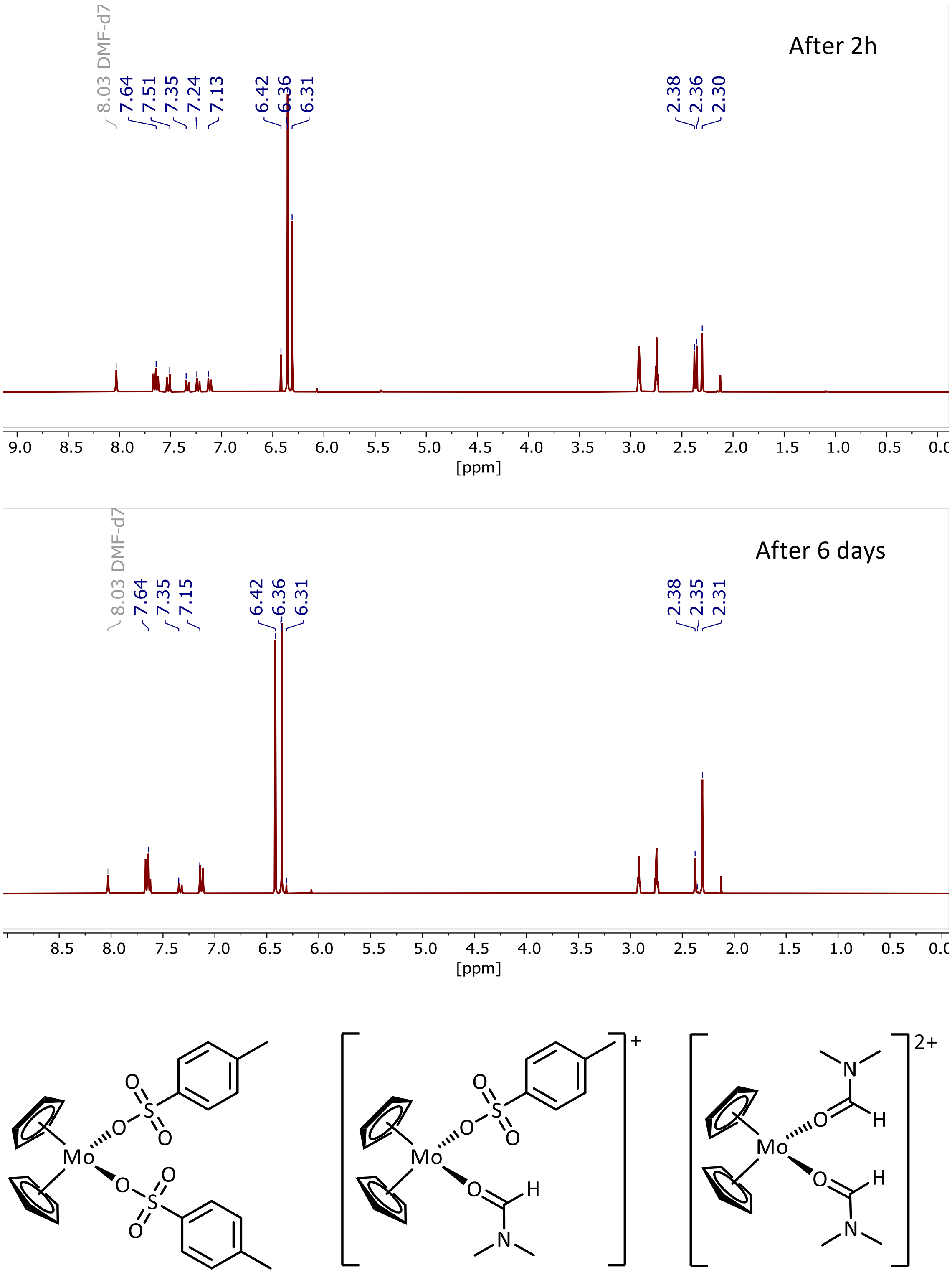

3

Figure S12. ^{1}H NMR spectra of **3** in DMF-d_7. Gradual substitution of the tosylates leads to three different species: Top spectrum after 2 h; bottom spectrum after 6 days. The signals corresponding to Cp-H clearly show this progress: 6.31 ppm corresponds to **3**, 6.36 ppm to the mono substituted [$Cp_2Mo(p$TsO)(DMF-d_7)]$^+$ and 6.42 ppm to the fully substituted [Cp_2Mo(DMF-$d_7)_2$]$^{2+}$.

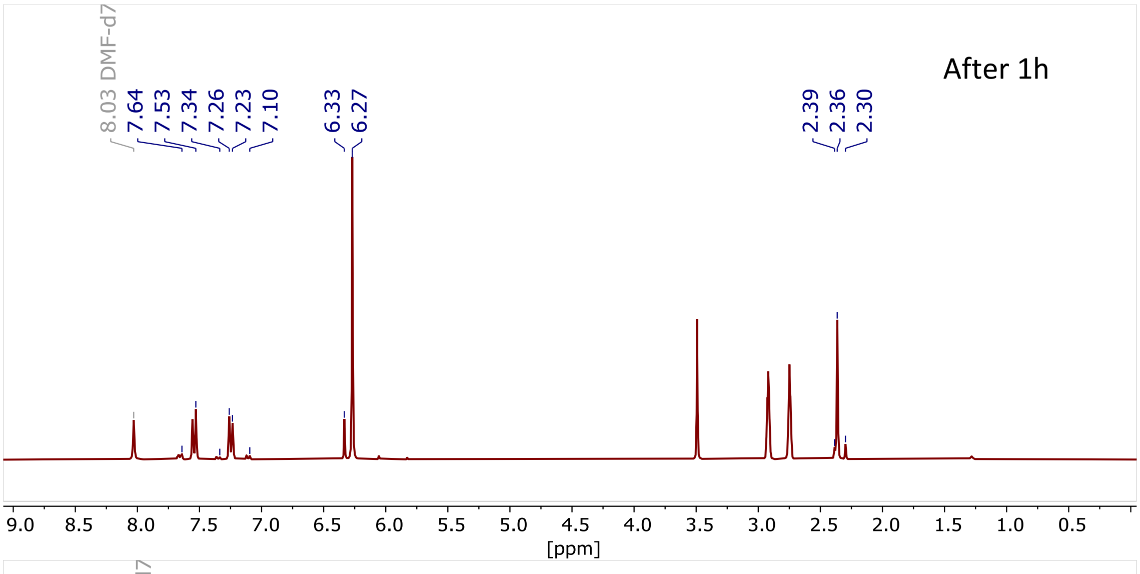

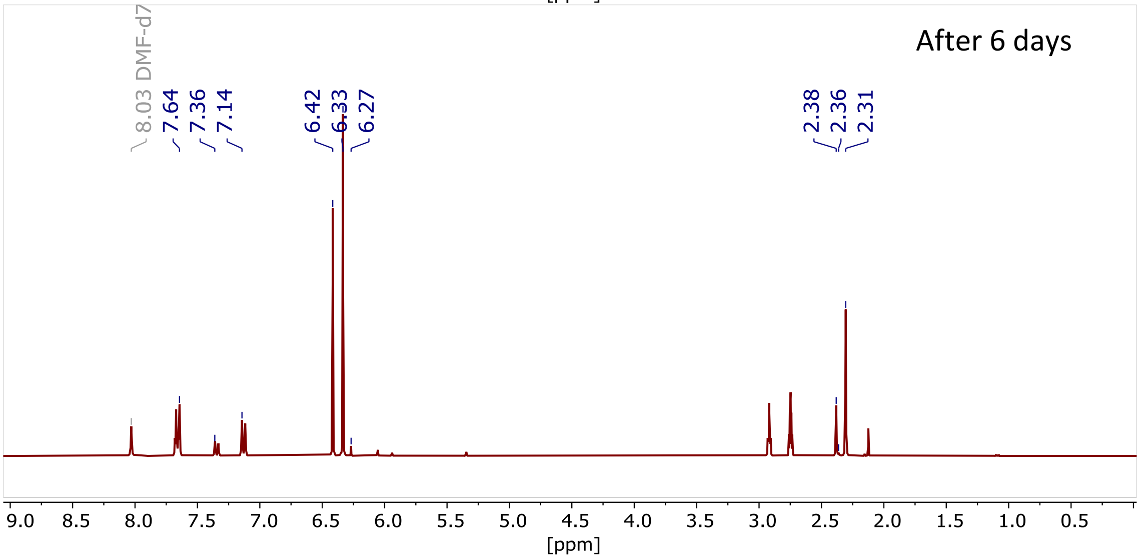

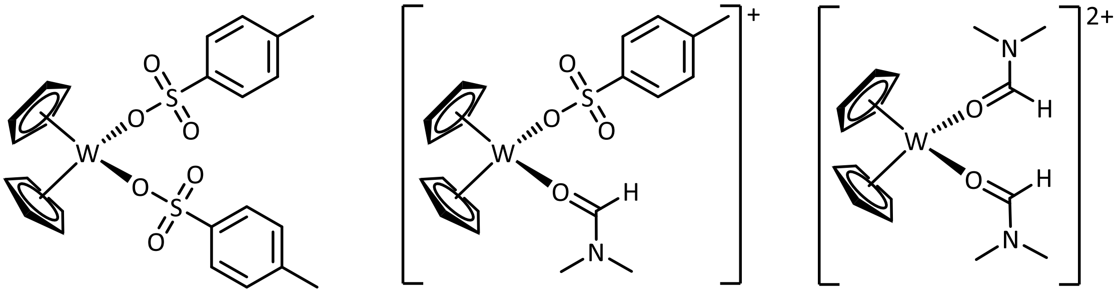

4

Figure S13. ^{1}H NMR spectra of **4** in DMF-d_7. Gradual substitution of the tosylates leads to three different species. Top spectrum after 1 h; bottom spectrum after 6 days. The signals corresponding to Cp-H clearly show this progress: 6.27 ppm corresponds to **4**, 6.33 ppm to the mono substituted $[Cp_2W(p\text{TsO})(\text{DMF-}d_7)]^+$ and 6.42 ppm to the fully substituted $[Cp_2W(\text{DMF-}d_7)_2]^{2+}$.

.

Comparison of Hydrolysis Products of 1a, 1b, 2a and 2b

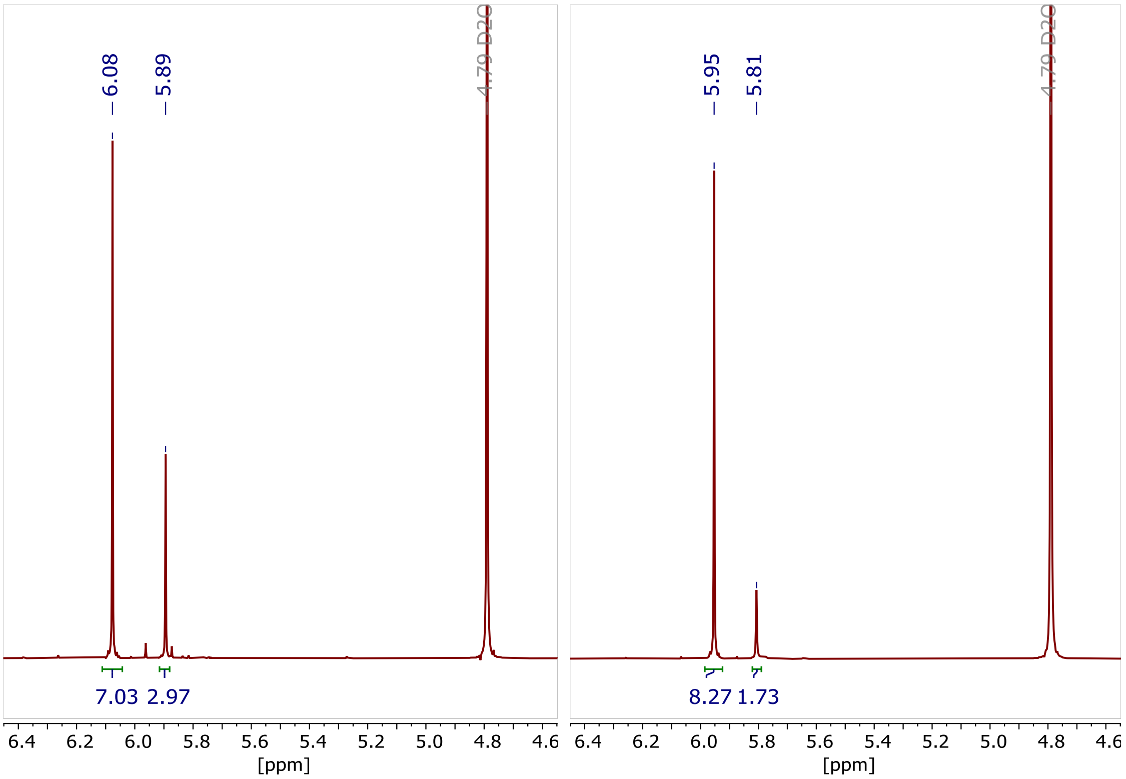

Figure S14. ^{1}H NMR spectra (Cp region) of hydrolyzed **1a** (left) and **1b** (right) in D_2O.

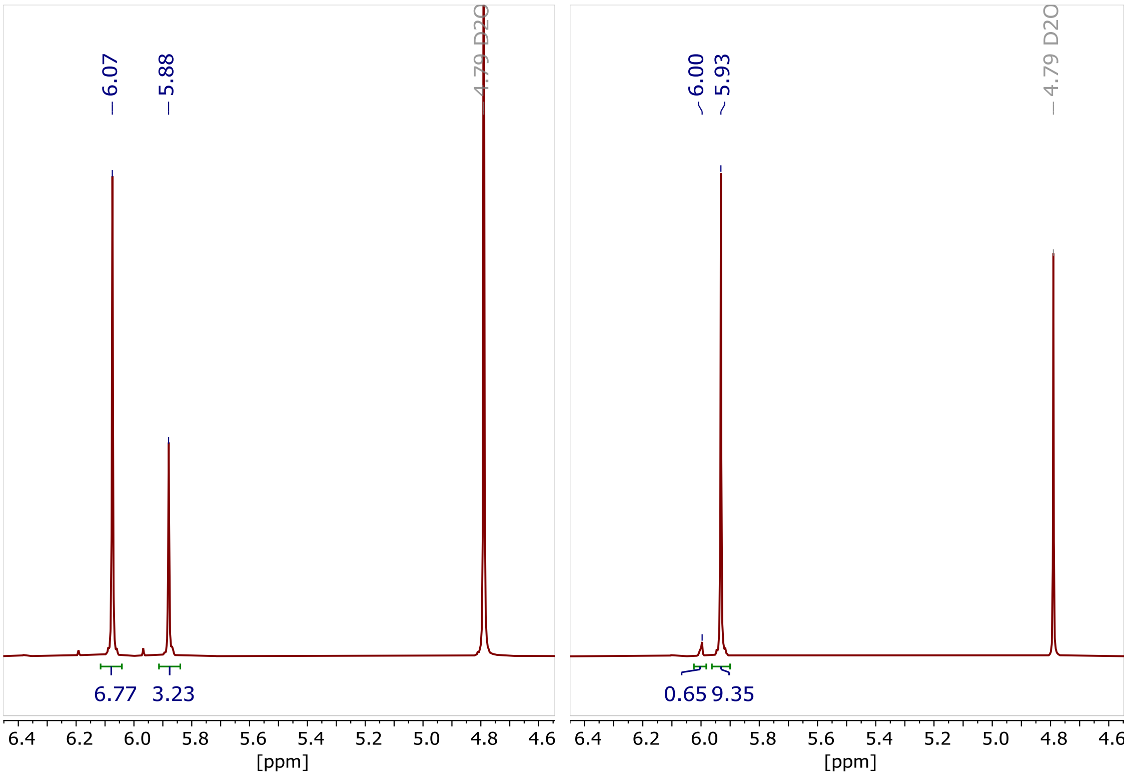

Figure S15. ^{1}H NMR spectra (Cp region) of hydrolyzed **2a** (left) and **2b** (right) in D_2O.

Hydrolysis Products of 1a, 1b, 2a and 2b at pD 6.55 in MOPS Buffer

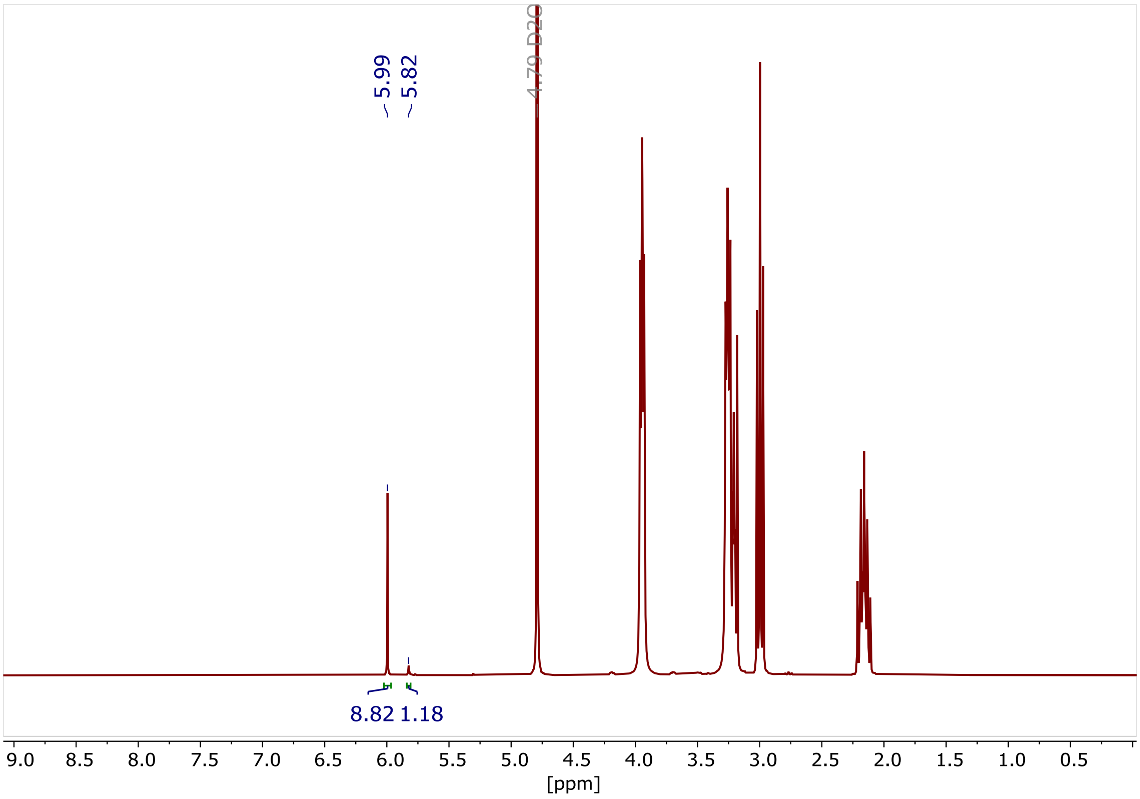

Figure S16: ^{1}H NMR spectrum of **1a** upon hydrolysis in 0.5 M MOPS in D_2O set to pD 6.55.

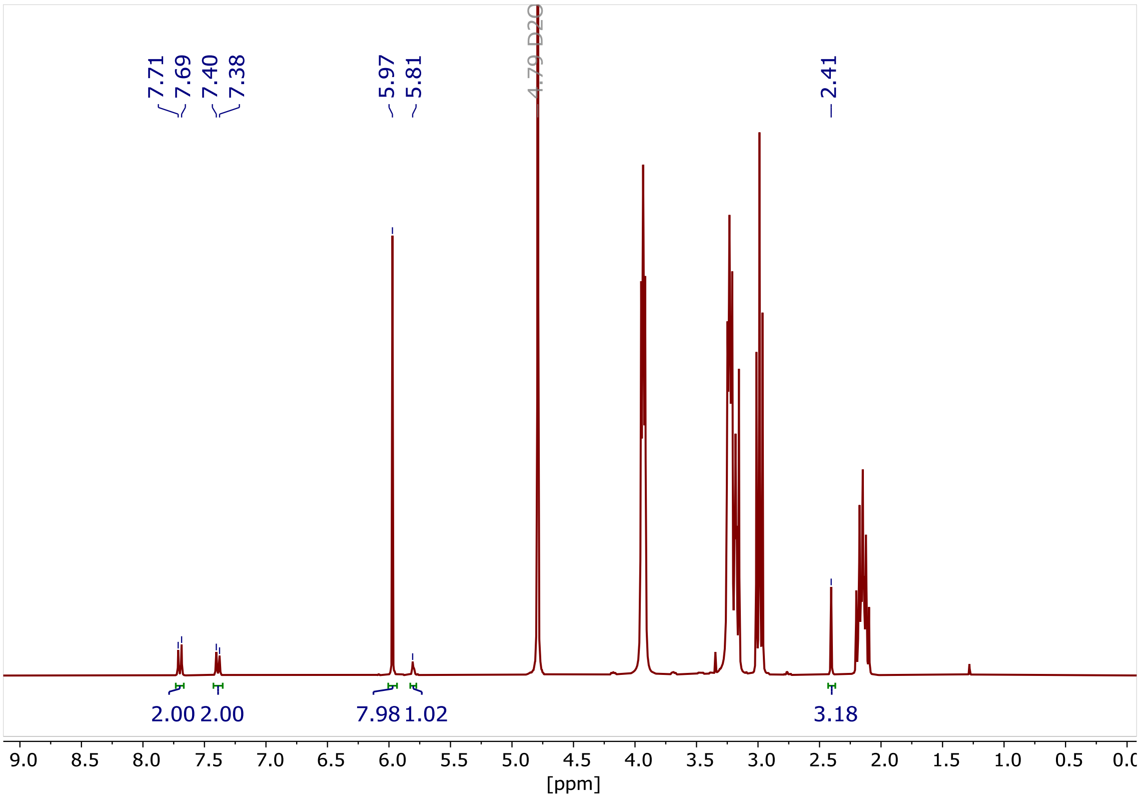

Figure S17: ^{1}H NMR spectrum of **1b** upon hydrolysis in 0.5 M MOPS in D_2O set to pD 6.55.

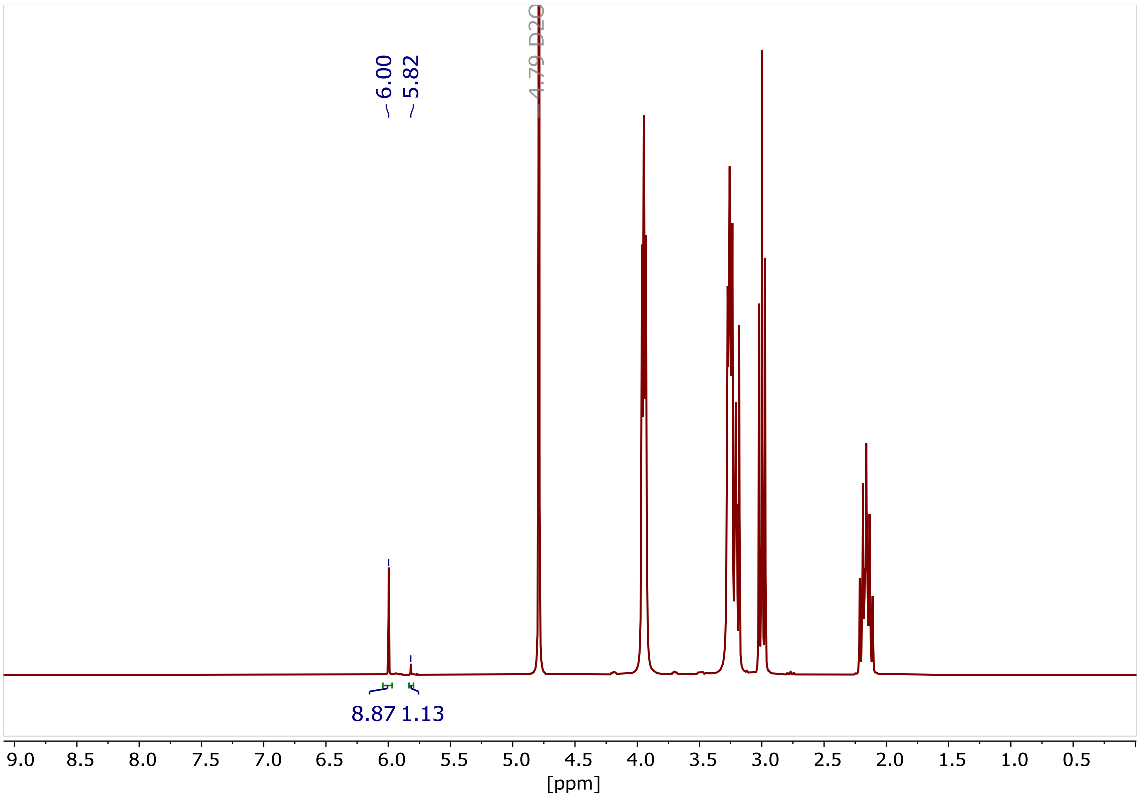

Figure S18: ^{1}H NMR spectrum of **2a** upon hydrolysis in 0.5 M MOPS in D_2O set to pD 6.55.

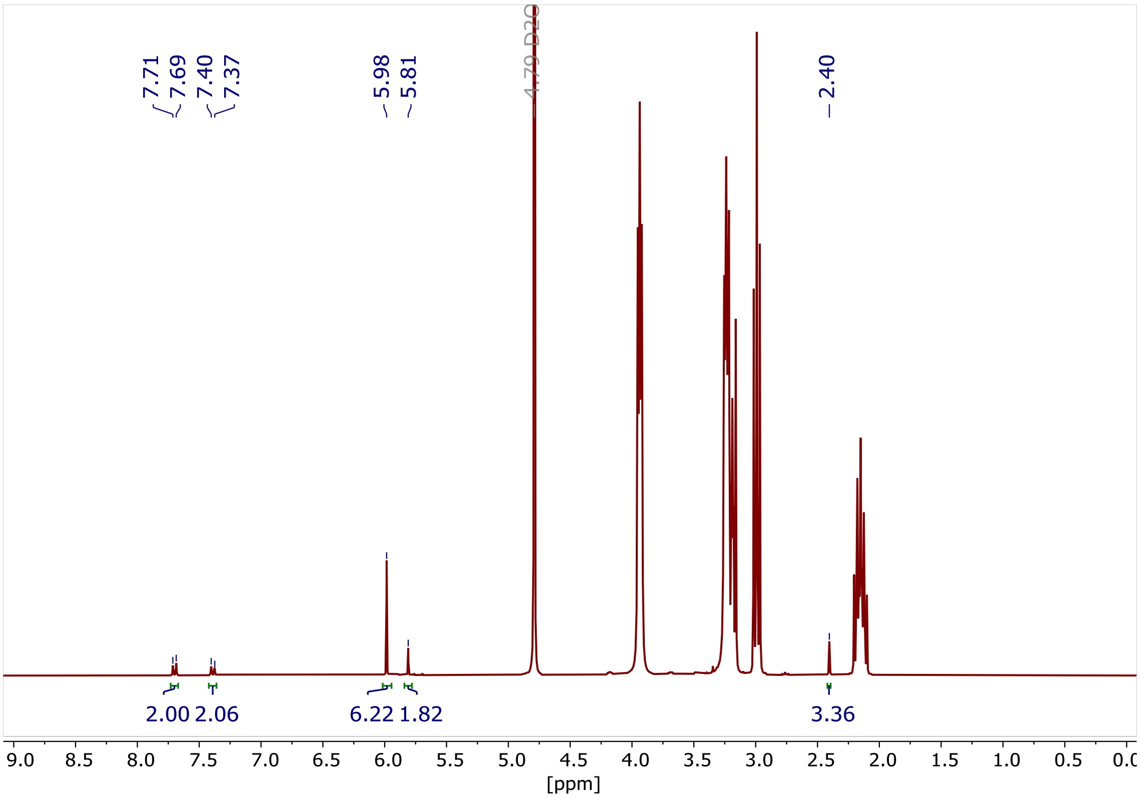

Figure S19: ^{1}H NMR spectrum of **2b** upon hydrolysis in 0.5 M MOPS in D_2O set to pD 6.55.

Hydrolysis of [Cp2Mo(*p*TsO)2] (3)

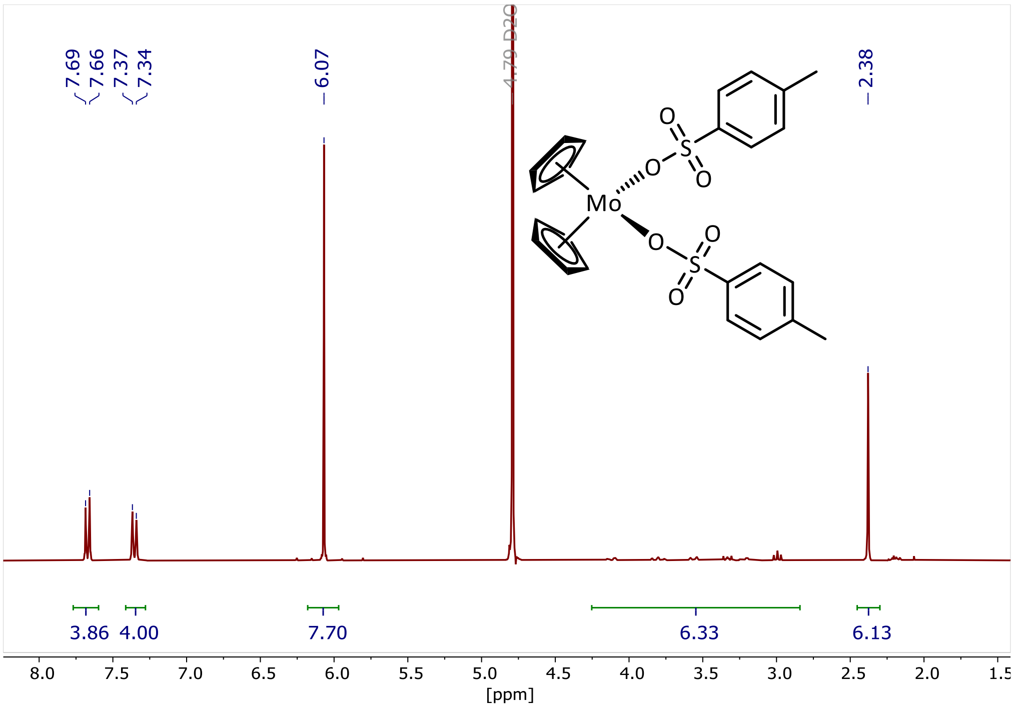

Figure S20. ^{1}H NMR spectrum of **3** upon hydrolysis at 80 °C in D_2O overnight. The signals at 7.67, 7.35 & 2.38 belong to ionic pTsO⁻. The signal at 6.07 belongs to Cp-H of hydrolyzed molybdocene. At 4.2 – 2.9 ppm residue belonging to free CpD is visible.

Hydrolysis of [Cp2W(pTsO)2] (4)

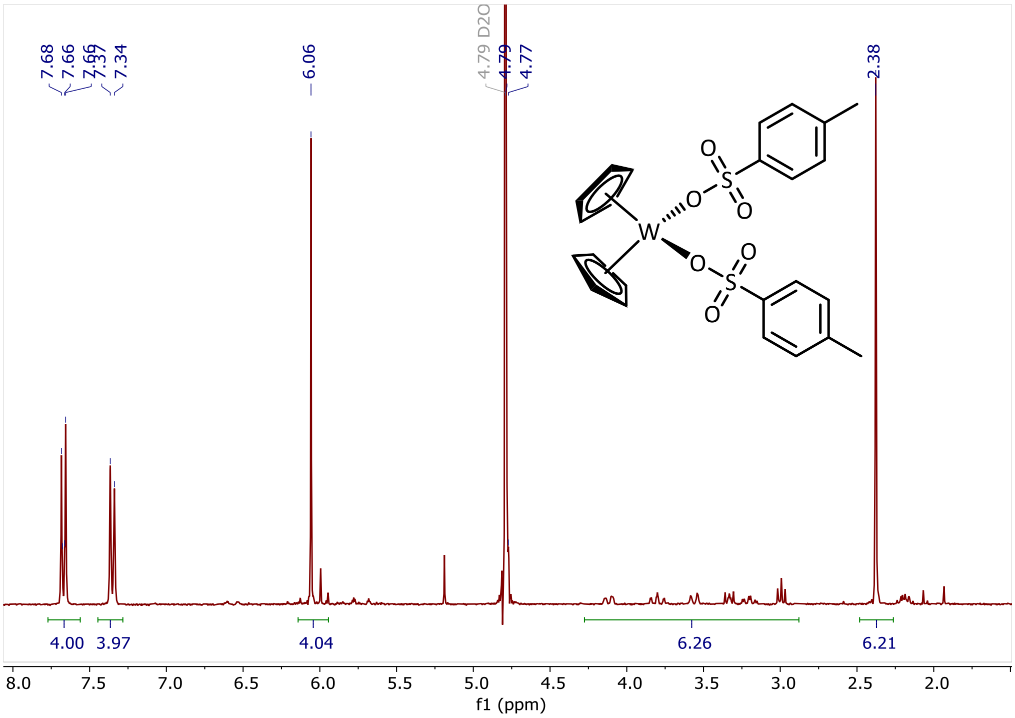

Figure S21. ^{1}H NMR spectrum of **4** upon hydrolysis at 80 °C in D_2O overnight. The signals at 7.67, 7.35 & 2.38 belong to ionic pTsO⁻. The signal at 6.06 belongs to Cp-H of hydrolyzed tungstocene. At 4.2 – 2.9 ppm residue belonging to free CpD is visible.

5 Crystal Structure Determination

General. The X-ray data collections were performed with a XtaLAB Synergy, Dualflex, HyPix-Arc 100 diffractometer at 100 K with Mo Kα radiation (λ= 0.71073 Å) for complex **2a** and with Cu Kα radiation (λ= 1.54184 Å) for complexes **3** and **4**. Data reduction, scaling and absorption corrections were performed using the *CrysAlisPro* software.[6] A numerical absorption correction based on gaussian integration over a multifaceted crystal model and an empirical absorption correction using spherical harmonics, implemented in SCALE3 ABSPACK scaling algorithm, were performed. The structures were solved with the ShelXT 2018/2[6] solution program using the intrinsic phasing solution method and by using Olex2[7] as the graphical interface. The models were refined with ShelXL 2019/3[8] using full matrix least squares minimization on F^2. All non-hydrogen atoms were refined anisotropically. Hydrogen atom positions were calculated geometrically and refined using a riding model. For **4**, SIMU restraints were used to model the disorder of the cyclopentadiene ligand. CCDC 2474413 – 2474415 contain the supplementary crystallographic data for this paper. This data can be obtained free of charge via http://www.ccdc.cam.ac.uk/ or from Cambridge Crystallographic Data Centre, 12 Union Road, Cambridge, CB2 1EZ, UK.

Table S3. Crystal data and structure refinement for **2a**, **3** and **4**.

Compound	**2a**	**3**	**4**
CCDC n°	2474413	2474415	2474414
Identification code	AD01_1	AD149	AD237
Empirical formula	$C_{10}H_{10}Cl_2W$	$C_{24}H_{24}O_6S_2Mo$	$C_{24}H_{24}O_6S_2W$
Formula weight	384.93	568.49	656.40
Temperature /K	99.9(2)	100.00(10)	100.00(15)
Crystal system	monoclinic	monoclinic	monoclinic
Space group	$P2_1/c$	$P2_1/c$	$P2_1/c$
a /Å	13.2843(2)	7.85040(10)	7.87300(10)
b /Å	12.88400(10)	26.5617(3)	26.5808(3)
c /Å	11.9437(2)	10.93040(10)	10.90380(10)
α /°	90	90	90
β /°	106.748(2)	94.6380(10)	94.8290(10)
γ /°	90	90	90
Volume /$Å^3$	1957.51(5)	2271.74(4)	2273.75(4)
Z	8	4	4
ρ_{calc} g/cm^3	2.612	1.662	1.918
μ /mm^{-1}	12.292	6.786	11.477
F(000)	1424.0	1160.0	1288.0
Crystal size /mm^3	0.19 × 0.16 × 0.06	0.14 × 0.02 × 0.01	0.15 × 0.13 × 0.03
Radiation	Mo Kα (λ=0.71073 Å)	Cu Kα (λ=1.54184 Å)	Cu Kα (λ = 1.54184 Å)
2Θ range for data collection/°	4.5 to 61.016	6.656 to 148.984	6.65 to 154.746
Index ranges	-18 ≤ h ≤ 18, -18 ≤ k ≤ 18, -17 ≤ l ≤ 17	-7 ≤ h ≤ 9, -32 ≤ k ≤ 33, -13 ≤ l ≤ 13	-9 ≤ h ≤ 8, -33 ≤ k ≤ 26, -13 ≤ l ≤ 13
Reflections collected	58628	24374	24910
Independent reflections	5974 [R_{int} = 0.0414, R_{sigma} = 0.0211]	4649 [R_{int} = 0.0382, R_{sigma} = 0.0307]	4814 [R_{int} = 0.0410, R_{sigma} = 0.0290]
Data / restraints / parameters	5974/0/235	4649/0/300	4814/30/300
Goodness-of-fit on F^2	1.042	1.034	1.129
Final R indexes [I ≥ 2σ(I)]	R_1 = 0.0171 wR_2 = 0.0387	R_1 = 0.0235, wR_2 = 0.0573	R_1 = 0.0368, wR_2 = 0.0902
Final R indexes [all data]	R_1 = 0.0210 wR_2 = 0.0398	R_1 = 0.0277, wR_2 = 0.0586	R_1 = 0.0396, wR_2 = 0.0918
Largest diff. peak/hole / e.$Å^{-3}$	1.47/-1.05	0.59/-0.57	3.28/-1.93

5.1. [Cp_2WCl_2] (2a)

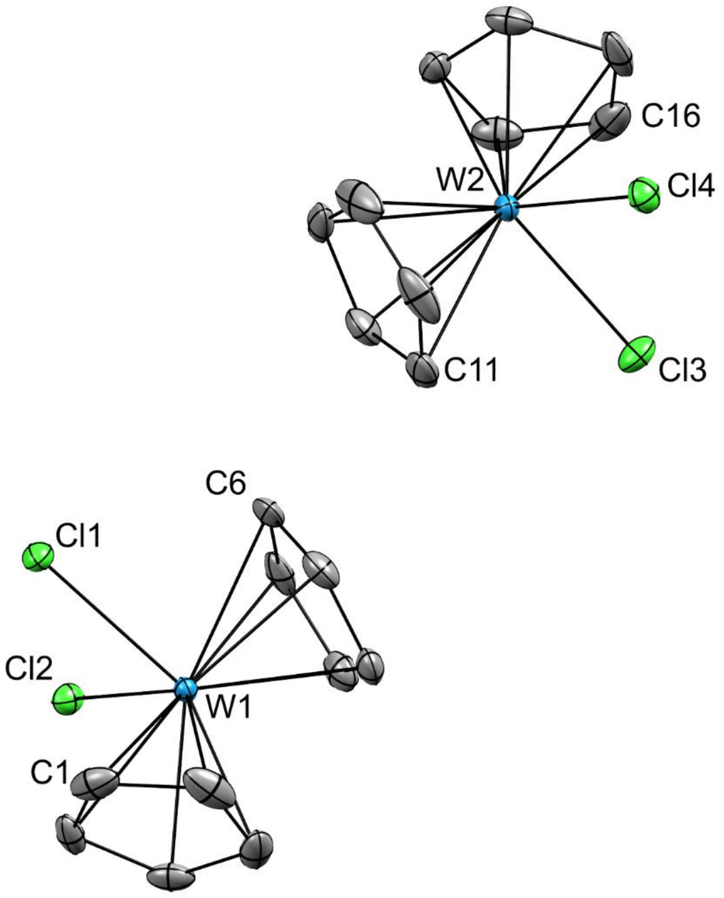

Figure S22. Molecular structure of **2a**. The probability ellipsoids are drawn at the 50% level. The H atoms were omitted for clarity.

There are two independent molecules in the asymmetric unit of **2a**. The complexes exhibit a typical bent metallocene structure, very similar to that of [Cp_2MoCl_2],[7] with W-Cl bond lengths between 2.4692(6) Å (W1-Cl2) and 2.4852(6) Å (W1-Cl1). One of the complex molecules has an almost eclipsed conformation of the Cp ring (C19-C18-C13-C14 7.1(3)°), the other molecule has a more staggered conformation (C2-C3-C8-C7 18.6(3)°).

Table S4. Selected bond lengths in Å for **2a**.

Atom	Atom	Length
W1	Cl1	2.4852(6)
W1	Cl2	2.4692(6)
W1	C4	2.263(3)
W1	C6	2.384(2)
W1	C8	2.239(2)
W1	C10	2.376(2)

Atom	Atom	Length
W2	Cl3	2.4708(6)
W2	Cl4	2.4711(6)
W2	C13	2.249(3)
W2	C15	2.379(3)
W2	C16	2.379(3)
W2	C18	2.239(3)

Table S5. Selected bond angles in ° for **2a**.

Atom	Atom	Atom	Angle
Cl2	W1	Cl1	80.82(2)
C1	W1	Cl1	77.03(7)
C1	W1	Cl2	109.02(8)
C1	W1	C10	171.42(11)
C7	W1	C1	113.98(11)

Atom	Atom	Atom	Angle
Cl3	W2	Cl4	80.48(2)
C11	W2	Cl3	77.38(7)
C11	W2	Cl4	94.90(7)
C11	W2	C20	170.75(10)
C17	W2	C11	128.41(10)

5.2. [$Cp_2Mo(p$TsO$)_2$] (3)

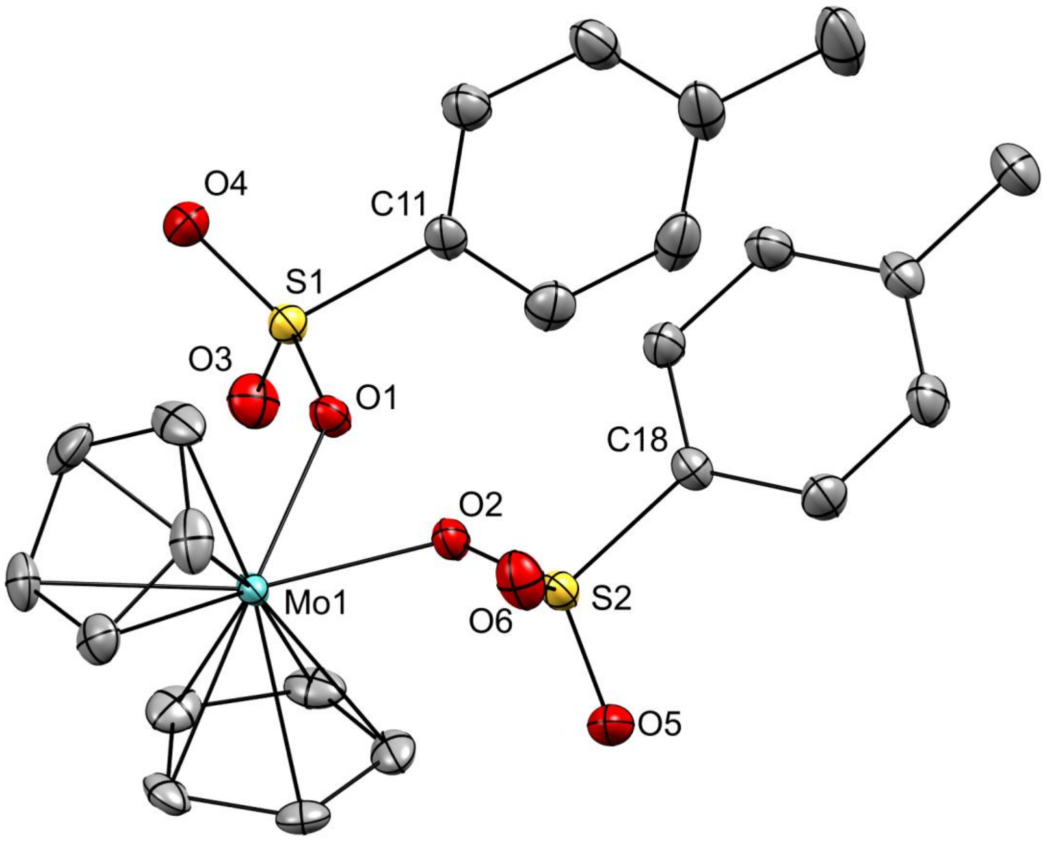

Figure S23. Molecular structure of **3**. The probability ellipsoids are drawn at 50% level. The H atoms were omitted for clarity

Complex **3** exhibits a typical bent metallocene structure, with two cyclopentadienyl ligands with Mo1-C bonds between 2.234(2) Å and 2.3709(19) Å. The two sulfonato ligands form almost a plane (S1-O1-Mo1-O2 178.05(15)°) with the two Cp below and above this plane. The Mo1-O1 and Mo1-O2 bond lengths (Mo1-O1 2.1256(13) Å and Mo1-O2 2.1229(13) Å) are similar to **4** (W1-O1 2.113(3) Å and W1-O2 2.114(3) Å).

Table S6. Selected bond lengths in Å for **3**.

Atom	Atom	Length	Atom	Atom	Length
Mo1	O1	2.1256(13)	S1	O3	1.4438(15)
Mo1	O2	2.1229(13)	S1	O4	1.4432(15)
Mo1	C1	2.3709(19)	S1	C11	1.771(2)
Mo1	C3	2.2498(19)	S2	O2	1.4856(14)
Mo1	C6	2.234(2)	S2	O5	1.4450(15)
Mo1	C9	2.3561(19)	S2	O6	1.4458(15)
S1	O1	1.4978(13)	S2	C18	1.7700(19)

Table S7. Selected bond angles in ° for **3**.

Atom	Atom	Atom	Angle	Atom	Atom	Atom	Angle
O1	Mo1	O2	71.22(5)	O1	S1	C11	99.55(8)
O1	Mo1	C1	77.77(6)	O3	S1	O1	111.85(8)
O1	Mo1	C3	133.28(7)	O3	S1	C11	108.53(9)
O1	Mo1	C6	81.68(7)	O4	S1	O1	111.94(8)
O1	Mo1	C9	139.61(6)	O4	S1	O3	115.11(9)
O2	Mo1	C2	134.56(7)	O4	S1	C11	108.61(9)
O2	Mo1	C5	78.02(6)	O2	S2	C18	100.83(8)
O2	Mo1	C8	139.39(7)	O5	S2	O2	112.40(9)
O2	Mo1	C10	81.22(6)	O5	S2	O6	114.13(9)
C3	Mo1	C8	75.78(8)	O5	S2	C18	108.21(9)
S1	O1	Mo1	138.82(8)	O6	S2	O2	112.30(9)
S2	O2	Mo1	142.14(8)	O6	S2	C18	107.91(9)

Table S8. Selected torsion angles in ° for **3**.

Atom	Atom	Atom	Atom	Angle
S1	O1	Mo1	O2	178.05(15)

5.3. [Cp_2W(*p*ToS)$_2$] (4)

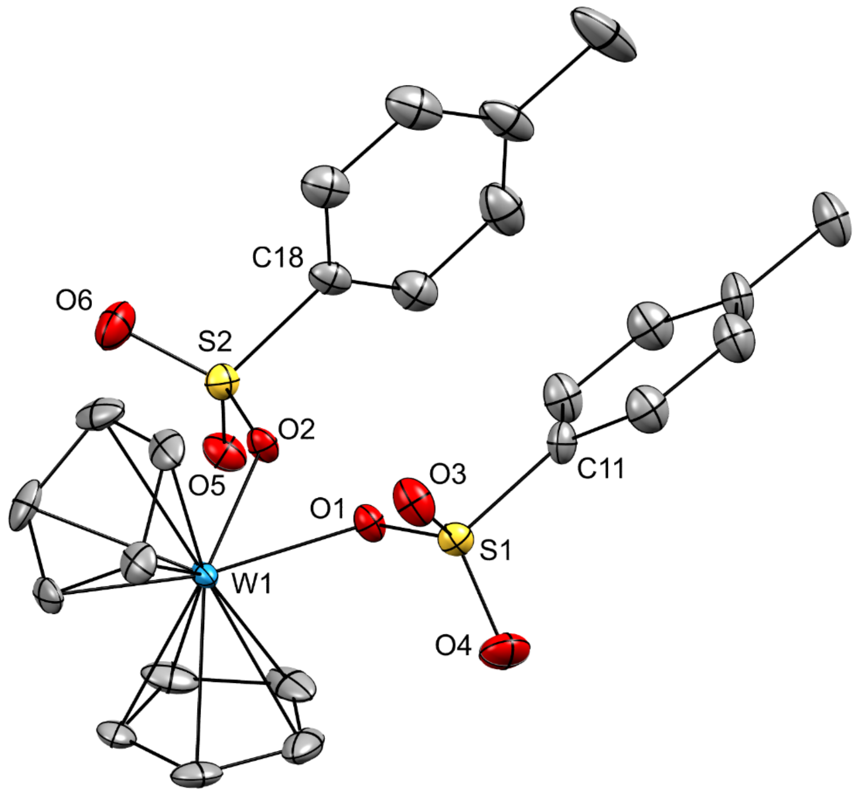

Figure S24. Molecular structure of **4.** The probability ellipsoids are drawn at the 40% level. The H atoms were omitted for clarity.

Similar to **3**, complex **4** exhibits a typical bent metallocene structure, with two cyclopentadienyl ligands with W1-C bonds between 2.235(5) Å and 2.366(5) Å. The two sulfonato ligands form almost a plane (S1-O1-W1-O2 172.6(4)°) with the two Cp's below and above this plane. The W1-O1 and W1-O2 bond lengths (W1-O1 2.113(3) Å and W1-O2 2.114(3) Å) are similar to the already reported tungstocene $[Cp_2W(trop)]^{+}OTs^{-}$ (W1-O1 2.105(5) Å).[8]

Table S9. Selected bond lengths in Å for **4**.

Atom	Atom	Length	Atom	Atom	Length
W1	O1	2.113(3)	S1	O3	1.443(4)
W1	O2	2.114(3)	S1	O4	1.437(4)
W1	C1	2.352(5)	S1	C11	1.770(5)
W1	C3	2.235(5)	S2	O2	1.502(3)
W1	C5	2.366(5)	S2	O5	1.444(4)
W1	C7	2.251(6)	S2	O6	1.440(4)
S1	O1	1.490(3)	S2	C18	1.764(5)

Table S10. Selected bond angles in ° for **4**.

Atom	Atom	Atom	Angle	Atom	Atom	Atom	Angle
O1	W1	O2	70.32(13)	O1	S1	C11	100.4(2)
O1	W1	C1	77.57(17)	O3	S1	O1	112.0(2)
O1	W1	C4	134.62(19)	O3	S1	C11	108.5(2)
O1	W1	C6	86.3(2)	O4	S1	O1	112.4(2)
O1	W1	C8	138.37(17)	O4	S1	O3	114.6(2)
O2	W1	C3	133.35(17)	O4	S1	C11	107.7(3)
O2	W1	C5	77.40(17)	O2	S2	C18	99.6(2)
O2	W1	C6	81.5(2)	O5	S2	O2	111.9(2)
O2	W1	C9	139.46(18)	O5	S2	C18	109.0(2)
C3	W1	C8	75.3(2)	O6	S2	O2	111.0(2)
S1	O1	W1	141.8(2)	O6	S2	O5	115.3(2)
S2	O2	W1	138.5(2)	O6	S2	C18	108.9(3)

Table S11. Selected torsion angles in ° for **4**.

Atom	Atom	Atom	Atom	Angle
S1	O1	W1	O2	172.6(4)

6 References

(1) Gluyas, J. B. G.; Brown, N. J.; Farmer, J. D.; Low, P. J. Optimised Syntheses of the Half-Sandwich Complexes FeCl(dppe)Cp*, FeCl(dppe)Cp, RuCl(dppe)Cp*, and RuCl(dppe)Cp. *Aust. J. Chem.* **2017**, *70* (1), 113.

(2) Luo, L.; Lanza, G.; Fragalà, I. L.; Stern, C. L.; Marks, T. J. Energetics of Metal−Ligand Multiple Bonds. A Combined Solution Thermochemical and ab Initio Quantum Chemical Study of M=O Bonding in Group 6 Metallocene Oxo Complexes. *J. Am. Chem. Soc.* **1998**, *120* (13), 3111–3122.

(3) Cooper, R. L.; Green, M. L. H. Some bis-π-cyclopentadienyl halides of molybdenum, tungsten, and rhenium. *J. Chem. Soc. A* **1967**, *0* (0), 1155–1160.

(4) Ren, J.-G.; Tomita, H.; Minato, M.; Osakada, K.; Ito, T. Syntheses, Structures, and Some Reactions of Di- *μ* -Hydroxo Dinuclear Complexes of Tungsten(IV) and Molybdenum(IV). *Chem. Lett.* **1994**, *23* (3), 637–640.

(5) Harris, D. C. *Exploring chemical analysis*; Freeman, 1997.

(6) Sheldrick, G. M. SHELXT - integrated space-group and crystal-structure determination. *Acta Crystallogr. A* **2015**, *71* (Pt 1), 3–8.

(7) Prout, K.; Cameron, T. S.; Forder, R. A.; Critchley, S. R.; Denton, B.; Rees, G. V. The crystal and molecular structures of bent bis-π-cyclopentadienyl–metal complexes: (a) bis-π-cyclopentadienyldibromorhenium(V) tetrafluoroborate, (b) bis-π-cyclopentadienyldichloromolybdenum(IV), (c) bis-π-cyclopentadienylhydroxomethylaminomolybdenum(IV) hexafluorophosphate, (d) bis-π-cyclopentadienylethylchloromolybdenum(IV), (e) bis-π-cyclopentadienyldichloroniobium(IV), (f) bis-π-cyclopentadienyldichloromolybdenum(V) tetrafluoroborate, (g) μ-oxo-bis[bis-π-cyclopentadienylchloroniobium(IV)] tetrafluoroborate, (h) bis-π-cyclopentadienyldichlorozirconium. *Acta Crystallogr. B* **1974**, *30* (10), 2290–2304.

(8) Minato, M.; Ren, J.-G.; Kasai, M.; Munakata, K.; Ito, T. Synthesis of molybdenocene(IV) and tungstenocene(IV) tropolonato complexes: Its derivative containing calix[4]arene moiety. *J. Organomet. Chem.* **2006**, *691* (3), 282–286.
